# ANNalog: generation of MedChem-similar molecules

**DOI:** 10.1186/s13321-026-01186-6

**Published:** 2026-04-15

**Authors:** Wei Dai, Jonathan D. Tyzack, Arianna Fornili, Chris de Graaf, Noel M. O’Boyle

**Affiliations:** 1 School of Physical and Chemical Sciences, Queen Mary University of London, London, E1 4NS UK; 2 Nxera Pharma, Steinmetz Building, Granta Park, Great Abington, Cambridge, CB21 6DG UK; 3 Structure Therapeutics, 601 Gateway Blvd, Suite 900, South San Francisco, CA 94080 USA; 4 EMBL’s European Bioinformatics Institute (EMBL-EBI), Wellcome Genome Campus, Hinxton, Cambridgeshire, CB10 1SD UK

**Keywords:** Generative modelling, Molecular generation, Scaffold hopping

## Abstract

Generative deep learning models have demonstrated significant potential in designing drug-like molecules. However, medicinal chemistry typically requires generating analogues that combine structural similarity with scaffold hopping, which is the replacement of molecular scaffolds while retaining biological relevance. To address this, we introduce ANNalog, a transformer-based sequence-to-sequence generative model trained on pairs of molecules extracted from the same bioactivity assay in a paper as recorded in ChEMBL33. The dataset was constructed based on the idea that molecules tested within the same assay can be considered analogues in medicinal chemistry space. Paired molecules were encoded as Simplified Molecular Input Line Entry System strings, and Levenshtein distance-guided alignment was applied to maximise intrapair string similarity; this preprocessing step was found to markedly enhance model performance. ANNalog has the ability to produce structurally similar analogues involving minor modifications, such as substituent replacements, as well as the ability to perform scaffold hopping, generating structurally distinct yet chemically relevant analogues. Scaffold-hopping capability was validated using manually curated molecule pairs and further confirmed through a case study involving orexin-2 receptor antagonists from patent literature. When the generation process was constrained using ANNalog’s prefix control feature, approximately 25% of the known scaffolds from the patent set were successfully recovered by the model, illustrating enhanced performance under user-guided conditions. Scientific Contribution: This study introduces ANNalog, a generative model trained using pairs of molecules synthesised and tested together within the same medicinal chemistry project. Unlike previous models trained on pairs of molecules selected according to similarity measures, ANNalog successfully generates not only structurally similar molecules but also diverse scaffold-hopping transformations that have precedent in the medicinal chemistry literature.

**Supplementary Information:**

The online version contains supplementary material available at 10.1186/s13321-026-01186-6.

## Introduction

In recent years, generative models based on deep learning have become valuable tools in drug discovery for their ability to rapidly generate new molecular structures. Broadly, these models can be categorised as either de novo generative methods or molecular optimisation approaches.

De novo generative models typically produce novel molecular structures without explicit reference to existing compounds, utilising architectures such as variational autoencoders (VAEs) [1–3], recurrent neural networks (RNNs) [4–6], and transformers [7–9]. Due to the inherent stochastic nature of these methods, techniques like reinforcement learning [10–12] and transfer learning [13–15] are frequently integrated to guide the generative process towards desirable chemical features.

In contrast, molecular optimisation models focus explicitly on modifying known molecular structures to optimise their chemical properties while retaining key biological features. A prominent example is the Mol2Mol transformer model developed by Tibo et al. [16], which was trained on a dataset of over 200 billion molecular pairs. This model explores local chemical space around input molecules, making it particularly suited to optimisation tasks within structurally related compound series.

In practical medicinal chemistry, however, chemists frequently combine local chemical-space exploration with scaffold hopping; this is the process of replacing the central structural core (scaffold) of biologically active molecules while retaining or enhancing their activity. Scaffold hopping is critically important because it enables access to structurally diverse chemical space, helps to navigate intellectual-property constraints [17] and can help migrate pharmacokinetic [18] or toxicity liabilities associated with the original scaffold [19].

To address this practical need, we introduce ANNalog, a transformer-based sequence-to-sequence (Seq2Seq) generative model explicitly designed to perform both local chemical space exploration and scaffold hopping within a unified framework. ANNalog is trained using pairs of molecules, selected without relying on computational structural similarity measurement. Instead, our model is based on a medicinal chemistry similarity definition proposed by O’Boyle et al. [20], where two molecules are considered similar if medicinal chemists would reasonably synthesise and evaluate them within the same drug discovery programme.

## Methodology

**Fig. 1:**
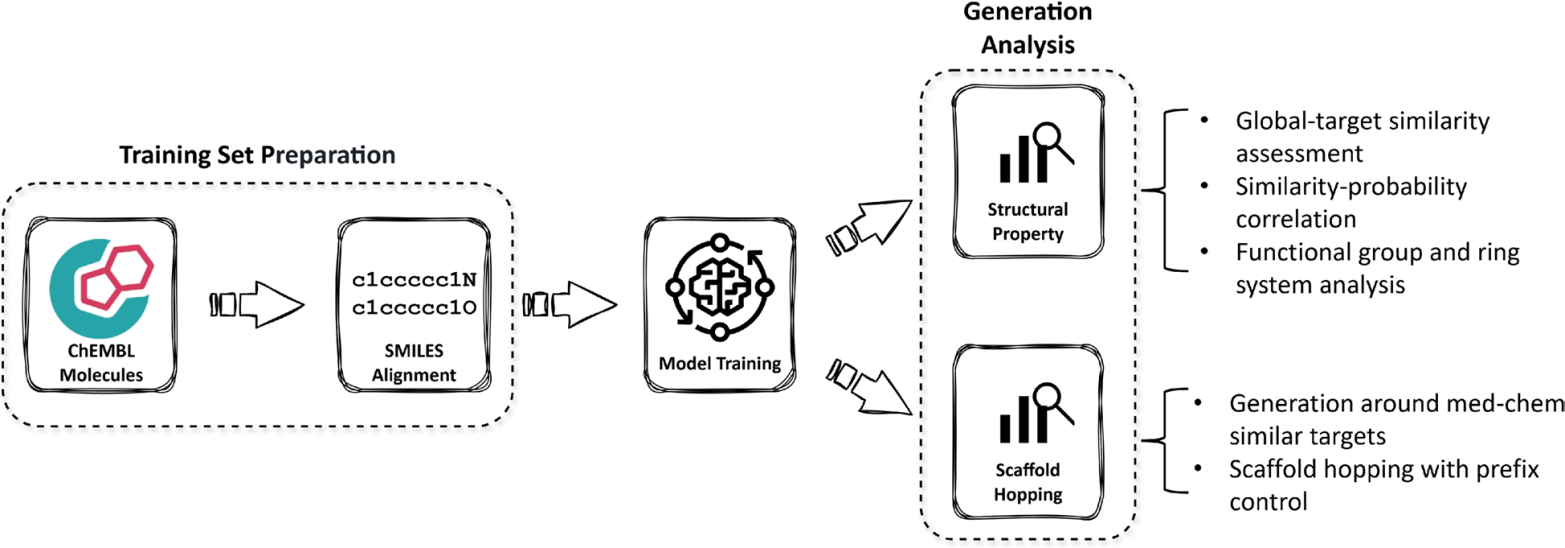
**ANNalog pipeline overview.** Schematic of the ANNalog pipeline showing training-set preparation (molecule extraction from ChEMBL33 and Levenshtein-guided SMILES alignment), Seq2Seq model training, and subsequent generative evaluation focusing on structural similarity and scaffold hopping

The general workflow of the ANNalog pipeline, from training set preparation through model training to generation analysis, is illustrated in Fig. 1. Based on the medicinal chemistry-driven similarity concept, pairs of molecules from the same assay were extracted from ChEMBL33 [21] with various filters applied. To simplify the process of learning structural transformations within molecule pairs, SMILES alignment was implemented by enumerating alternative SMILES representations for each molecule and selecting the SMILES pair that minimised the Levenshtein distance [22]. Additionally, four diverse SMILES representations for each molecular structure were generated as data augmentation, enhancing model generalisation across syntactic variations. The generative capability of ANNalog was then rigorously evaluated, specifically assessing the structural similarity to input ligands and scaffold hopping performance. Scaffold hopping quality was validated using manually curated molecule pairs and scaffold transformations derived from patent literature, while generative performance was benchmarked against the previously published Mol2Mol transformer model [16].

### Molecule-pairs set preparation

Assays were selected from the ChEMBL33 database following criteria established by O’Boyle et al. [20], ensuring each assay contained between 8 and 25 molecules and reported activity data types including IC_50_, pIC_50_, Ki, pKi, EC_50_, and pEC_50_. Only binding (B) and functional (F) assays were included, due to their relevance in evaluating molecular similarity.

Molecules within assays underwent additional filtering to ensure quality and relevance. All molecular structures were represented using ChEMBL parent structures (parent SMILES), which are standardised by ChEMBL to remove salts and solvents. An additional RDKit-based check was performed to confirm that salt and solvent components were not present in the final training data. Molecules considered "popular," such as approved drugs, those listed in Wikipedia, or cited in more than five publications, were excluded to reduce the chance of including molecules that are not similar to the others tested (such as internal standards). Further exclusions included peptides, isotopes, molecules exceeding a molecular weight of 600 Da, molecules containing atoms outside the set {C, N, H, O, S, F, Cl, Br}, those with more than seven rotatable bonds, and SMILES strings exceeding 100 tokens after tokenisation.

From an initial collection of 24,139 ChEMBL documents, 31,445 assays were identified. Within each assay, molecules were systematically paired, resulting in a total of 618,915 unique molecule pairs, constituting the molecule-pairs set, with up to 30 pairs extracted per individual document. This cap reduces over-representation from documents containing many retained assays. For each document, all candidate pairs across retained assays were enumerated and deduplicated; if more than 30 unique pairs remained, 30 pairs were randomly sampled without replacement. Molecule pairs differing solely in stereochemistry, including those cases where scaffold variation arose exclusively from stereochemical differences, were excluded. This filtration step ensured that the model would preserve existing stereochemical configurations rather than generate trivial permutations. Following filtration, 608,642 molecule pairs remained, represented as canonical SMILES. From this filtered collection, 80% (486,916 pairs) were randomly selected to create the training set for the sequence-to-sequence model, while the remaining 20% was evenly divided into a validation set and an internal test set using a fixed random seed of 42. The structural similarity distributions across the splits are shown in Supplementary Figure S1. These internal splits were used to monitor negative log-likelihood during training and for model selection. An additional analysis was performed based on Bemis–Murcko scaffolds, identifying that within the training set, 321,494 molecule pairs (66%) contained distinct scaffolds. To benchmark the quality of generated molecules on an independent dataset, an evaluation set derived from the later ChEMBL35 release was used for downstream analyses.

### Model implementation and sampling

#### Seq2Seq model training

ANNalog is a transformer sequence-to-sequence architecture adapted from Trevett’s PyTorch implementation [23], and previously used for molecular generation [24]. It comprises three encoder layers and three decoder layers. Each layer employs multi-head attention (eight heads; model width 256) followed by a position-wise feed-forward block (inner width 512). Positional embeddings encode token order. SMILES strings are tokenised with a custom tokeniser and mapped to a shared vocabulary that includes start, end, and padding symbols.

**Fig. 2:**
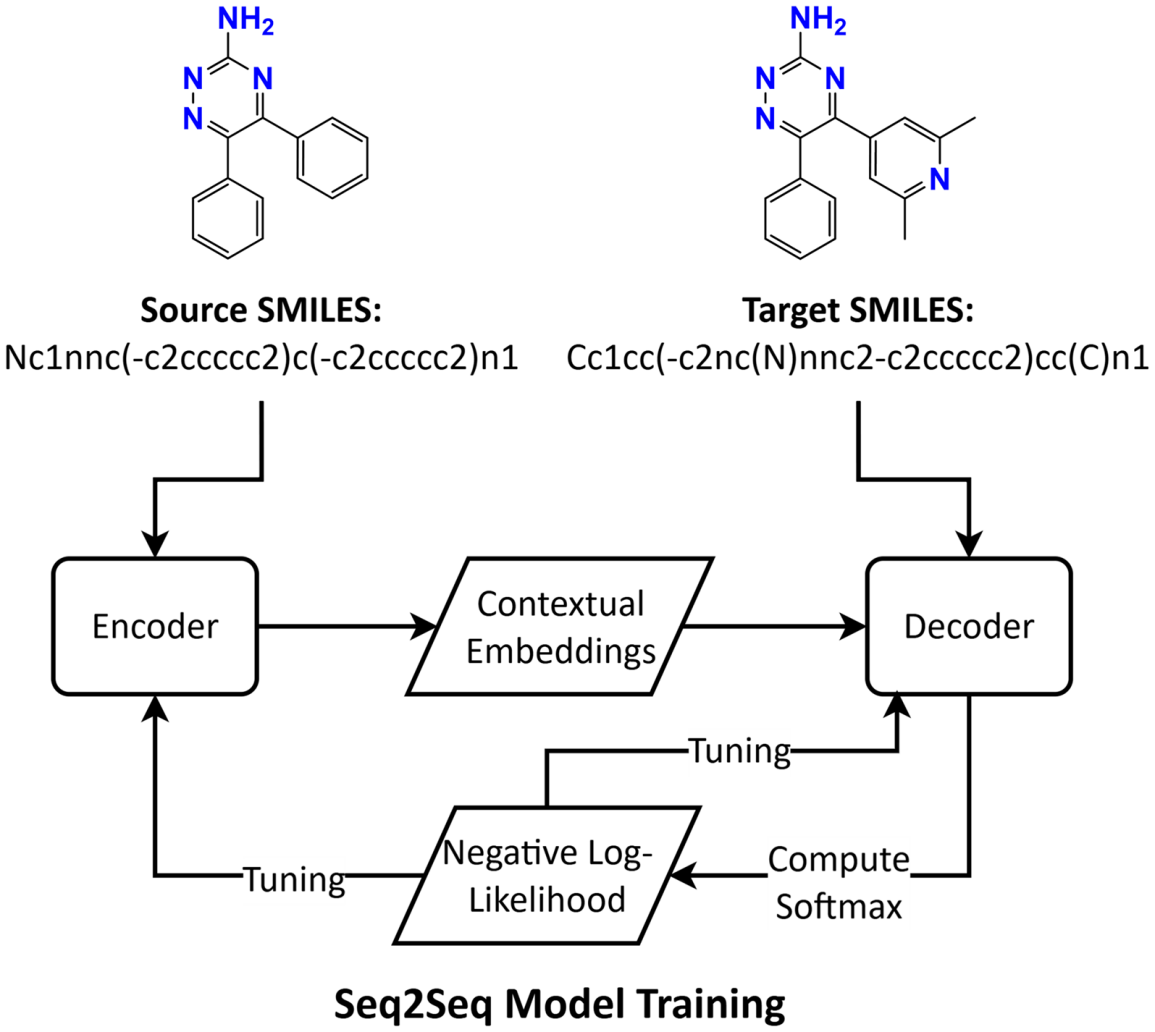
**Training workflow of the transformer Seq2Seq model for SMILES.** The model encodes the source SMILES into contextual embeddings (one representation per source token), receives the target sequence shifted by one position, and uses attention to predict the next token at each step. Decoder scores over the vocabulary are converted to probabilities, per-token negative log-likelihoods are accumulated across the sequence while padding is ignored, and back-propagation updates parameters in both the encoder and the decoder

The training workflow is shown in Fig. 2. For an illustrative training set pair indicated as source and target SMILES, the model tokenises and embeds the source SMILES and processes it with the encoder to produce a sequence of contextual embeddings, that is, vector representations for each source token that reflect both the token itself and its role within the full string (for example, relationships created by rings, branches, and bond markers). The target SMILES is also tokenised. During training, the decoder is provided with the target sequence shifted by one position, so that at each step it receives the known prefix and must predict the next token. Using attention, the model compares the target prefix with the encoded source representations and assigns a score to every token in the vocabulary. These scores are converted into probabilities using a standard softmax operation.

After converting the decoder’s scores (logits) $z_{t}$ to probabilities with a softmax over the vocabulary $V$ (the set of tokens the model can output, with size $\left| V \right|$), a categorical distribution $p_{t} \left( \cdot \right)$ over $V$ at step $t$ is obtained ($\left( \cdot \right)$ means "for any token").

The distribution is given by:

$$p_{t} \left( v \right) = {\mathrm{softmax}}\left( {z_{t} } \right)_{v} = \frac{{{\mathrm{exp}}\left( {z_{t,v} } \right)}}{{\mathop \sum \nolimits_{u \in V} {\mathrm{exp}}\left( {z_{t,u} } \right)}}$$

for $v \in V$, where $z_{t} \in { mathbb{R}}^{\left| V \right|}$ is the vector of logits at step $t$ (one unnormalised score per token), $p_{t} \left( v \right)$ denotes the probability assigned to token $v$, and the denominator sums over $u \in V$.

For a source sequence $x = \left( {x_{1} , \ldots ,x_{{T_{x} }} } \right)$ and a target sequence $y = \left( {y_{1} , \ldots ,y_{{T_{y} }} } \right)$ with start-of-sequence token $y_{1} = \left\langle {{\mathrm{sos}}} \right\rangle$, the same distribution can be written as $p_{t} \left( v \right) = P_{\theta } \left( {y_{t} = v{ mid }y_{ < t} ,x} \right)$, where $\theta$ are all trainable parameters of the encoder-decoder and $y_{ < t} = \left( {y_{1} , \ldots ,y_{t - 1} } \right)$ is the already known target prefix. Let the target token at step $t$ be $y_{t}$ and define the padding mask $m_{t} = 1\left[ {y_{t} \ne \left\langle {{\mathrm{pad}}} \right\rangle } \right]$, where $1\left[ \cdot \right]$ equals 1 if the condition holds and 0 otherwise; the per-token masked negative log-likelihood is $l_{t} = - m_{t} {\mathrm{log}}p_{t} \left( {y_{t} } \right)$, which penalises assigning low probability to the correct token and contributes zero when the position is padding (here ${\mathrm{log}}$ denotes the natural logarithm). The sequence-level objective for one example is:

$$L_{{{\mathrm{seq}}}} = \mathop \sum \limits_{t = 2}^{{T_{y} }} l_{t} = - \mathop \sum \limits_{t = 2}^{{T_{y} }} m_{t} {\mathrm{log}}P_{\theta } \left( {y_{t} { mid }y_{ < t} ,x} \right)$$

starting at $t = 2$ because $y_{1} = \left\langle {{\mathrm{sos}}} \right\rangle$ is given rather than predicted (teacher forcing).

Gradients $\nabla_{\theta } L_{{{\mathrm{seq}}}}$ are back-propagated through the decoder and, via cross-attention, into the encoder so that both parts update to reduce the loss; in practice, training uses mini-batches of size 64 and the Adam optimiser.

#### SMILES sampling and tuning

ANNalog implements three SMILES sampling methods, each offering different trade-offs between diversity and structural similarity:

- *Greedy search* [25] Generates molecules by sequentially selecting the token with the highest probability at each decoding step; equivalent to beam search with a beam width of 1.
- *Beam search (beam)* [26] Expands multiple candidate sequences simultaneously, retaining the top-*k* sequences at each step based on cumulative probability; *k* denotes the beam width.
- *Multinomial sampling (sampling)* [27] Introduces stochasticity through temperature-scaled probabilistic token selection (temperature = 1.2, applied consistently across all tasks), thereby promoting greater chemical diversity in the generated outputs.

ANNalog provides two optional tools that allow users to guide and constrain the SMILES sampling process. These mechanisms can be enabled or disabled depending on the specific application:

- *Prefix Control* This mechanism ensures that the generated SMILES begin with a user-defined prefix, allowing partial structural constraints to be enforced during generation. The user specifies a set of initial SMILES characters to be fixed at the beginning of the sequence. These characters are tokenised, and the model treats them as already generated before proceeding with the rest of the molecule.
- *Invalid Filtering* This mechanism improves the validity of generated outputs by removing partial sequences that cannot result in valid SMILES. It is implemented using the *partialsmiles* library [28], which evaluates each intermediate sequence during generation. After each token is added, the current partial SMILES is checked to determine whether it can be completed into a valid molecule. If not, the sequence is discarded from further expansion.

### Model performance assessment

#### Effect of SMILES alignment

To emphasise the structural differences between training set pairs and enhance the model’s ability to learn SMILES-level transformations, SMILES alignment was applied as a preprocessing strategy. The objective of SMILES alignment is to maximise character-level correspondence between paired SMILES strings, thereby reducing irrelevant sequence divergence and enabling the model to more effectively focus on meaningful structural changes. In this study, alignment was performed using Levenshtein distance [22], which measures similarity by calculating the minimum number of single-character edits (insertions, deletions, or substitutions) required to transform one SMILES string into another.

Three datasets were constructed from the molecule-pairs set:

- *Canonical dataset* Consisting of canonical SMILES pairs.
- *Randomised dataset* Comprising pairs with randomly shuffled atom indices, serving as a benchmark to assess the impact of atom-index randomisation.
- *Levenshtein-aligned (Lev_aligned) dataset* Each canonical SMILES was paired with the randomised SMILES of the paired molecule that minimised the Levenshtein distance among 10,000 candidates.

Each molecule pair was represented bidirectionally (A → B and B → A). Three independent Seq2Seq models were trained separately on these datasets using identical hyperparameters and configurations for 100 training epochs. Model loss curves are provided in the Supplementary Figure S2.

Model performance was evaluated using greedy decoding, generating one SMILES per input. To assess the robustness of the model to different SMILES representations, each model was evaluated using two input formats: RDKit [29] canonicalised SMILES, and also randomly shuffled SMILES representing the same molecule. The following metrics were used for evaluation:

- *Invalid rate* The proportion of generated outputs that cannot be parsed as valid SMILES by RDKit. In this experiment, the invalid filtering option was set to off during generation, so no partial sequence pruning was performed during decoding.
- *Structural similarity* Computed as the Tanimoto coefficient between the Morgan fingerprints (radius 2, bit length 16,384) [30] of the generated SMILES and the corresponding input source or target molecule.

#### Data augmentation with SMILES alignment

To improve the model’s robustness and its ability to interpret non-canonical representations, the dataset was further expanded to expose the model to multiple SMILES forms for each molecule. Passing varied SMILES representations of the same molecular structure allows the model to generalise across syntactic variations, while different alignments of source–target pairs highlight molecular differences from multiple perspectives. Based on this rationale, an extended dataset (Lev_extended) was constructed using Levenshtein alignment.

For each molecular pair (molecule A ↔ molecule B), five SMILES representations of molecule A (one canonical and four randomised variants) were generated by randomly shuffling atom indices using RDKit. In parallel, up to 10,000 randomised SMILES representations were independently generated for molecule B. Each SMILES of molecule A was individually aligned against all randomised SMILES of molecule B using Levenshtein distance, and the best-matching SMILES of molecule B was selected. The same procedure was applied in reverse, aligning SMILES of molecule B to randomised forms of molecule A. In total, ten optimized SMILES pairs were obtained per original molecular pair (five in each direction) and compiled to form the Lev_extended dataset.

A single Seq2Seq model was trained on the Lev_extended dataset for 100 epochs (loss curves provided in Supplementary Figure S2), evaluated using the same benchmarks and procedures described in the previous methods section.

#### Structural properties of generated molecules

An independent test set of 1000 assays was extracted from a later release of ChEMBL, ChEMBL35, and filtered identically to the molecule-pairs set to exclude any overlap with the ChEMBL33-derived training set and remove all molecules present therein. For each assay, one molecule was randomly selected as input to ANNalog, which was used to generate sets of 50, 100, 250, 500 and 1000 SMILES per input using both the beam search and multinomial sampling methods. Three analyses were subsequently performed:

- *Global-target similarity assessment* Each generated SMILES was compared to the assay’s “global target” (the molecule within the same assay exhibiting the highest Tanimoto similarity) to evaluate structural relevance.
- *Similarity–probability correlation* The relationship between each candidate’s Tanimoto similarity to the input molecule and its negative log-likelihood under ANNalog was analysed, thereby assessing how likelihood varies with structural proximity.
- *Functional group and ring system analysis* Generated molecules were analysed by focusing on the functional groups and ring systems they contained: functional groups were identified using Ertl’s methodology as implemented in RDKit [31] and ring systems were extracted via OpenBabel following O’Boyle’s approach [32].

### Ability to scaffold hop

#### Generation of neighbours around MedChem-similar targets

From the 1000-assay cohort, 108 manually-selected molecule pairs were designated as explicit source–target instances. These molecule pairs were specifically chosen to include only core scaffold and ring-system transformations, such as heterocycle interconversions, ring expansions and contractions, and intramolecular cyclisation. Peripheral substituent exchanges (e.g., halogen or CF₃ substitutions) and structural insertions or deletions exceeding 30% of the total heavy-atom count were excluded. Additionally, these molecule pairs exhibit Tanimoto similarity values between 0.4 and 0.5 based on Morgan fingerprints, reflecting differences in their core scaffolds. The complete list of molecule pairs, represented as RDKit canonicalised SMILES, is provided in Supplementary Table T1.

Three evaluations were conducted:

- *Probability test* This tested the likelihood of generating each target SMILES from the same source input under two models, ANNalog and Mol2Mol, quantified by negative log-likelihood. The cumulative negative log-likelihood of each target SMILES conditioned on its corresponding source SMILES was calculated by summing the character-wise negative log-likelihood values obtained for ANNalog and Mol2Mol. This assessment was performed across three distinct SMILES representations of each source–target pair: canonicalised SMILES pairs, randomised SMILES pairs, and aligned SMILES pairs.
- *Neighbour-around-target test* This analysis compared ANNalog and Mol2Mol when given the same source input, with Mol2Mol included as a reference model for local analogue generation and expected to preferentially generate near neighbours with high similarity to the source molecule. First, an illustrative chemical-space visualisation was performed for one representative source–target pair, depicting spatial relationships between the source, target, and generated neighbours. Morgan fingerprints of these molecules were projected into two-dimensional space using t-distributed stochastic neighbour embedding (t-SNE) with the Jaccard metric (perplexity 15, early exaggeration 12.0, random state 42). Subsequently, an overview analysis was conducted across all source–target pairs. Here, ANNalog and Mol2Mol each generated up to 100 unique molecules per source using the beam search method, and pairwise Tanimoto similarities (Morgan fingerprints, radius 2, 16,384 bits) between generated molecules and their targets were calculated. For each pair, the ten generated molecules exhibiting the highest similarity to the target were identified, and their model origin (ANNalog or Mol2Mol) was recorded.

### Scaffold hopping with prefix control

To evaluate the scaffold hopping ability of the ANNalog model, a case study was conducted using a dataset of orexin-2 receptor antagonists. Orexin receptors, particularly the OX2 subtype, are established therapeutic targets for the treatment of sleep disorders [33, 34]. In this study, a collection of 380 octahydropyrrolo[3,4-c]pyrrole OX2 receptor antagonists was extracted from the patent US11059828B2 [35]. These patent-derived exemplified compounds can be regarded as structural analogues. Among the 380 molecules, 121 unique Bemis–Murcko scaffolds were identified and used to assess scaffold-level diversity.

The ANNalog model was evaluated on this dataset to assess its ability to generate novel scaffolds distinct from the input structures. The Mol2Mol model was used as reference. Performance was quantified using the scaffold recall rate, defined as the proportion of unique scaffolds (excluding the input scaffold) among the remaining 120 scaffolds that were recovered in the set of molecules generated for each input. For each of the 380 input molecules, ANNalog generated up to 5000 SMILES using both the beam search and multinomial sampling methods. For the Mol2Mol model, only beam search was applied, as this approach is recommended as the default decoding strategy in the original publication describing the model.

To mimic real-world applications in medicinal chemistry, prefix control and invalid filtering were activated. Prefix control was used to preserve a key substructure, the pyrrolo[3,4-c]pyrrole core, at the beginning of each generated molecule. Each input molecule’s SMILES was first subjected to atom index randomisation using RDKit, and the SMILES variant that placed the pyrrolo[3,4-c]pyrrole substructure at the start of the string was selected. If multiple SMILES variants satisfied this criterion, one variant was selected at random and used consistently for all subsequent experiments for that molecule. This prefix region was tokenised, and the prefix control mechanism ensured that the model treated these tokens as fixed during the generation process.

To isolate the effect of prefix control on scaffold recall, an additional comparison was conducted using the same prefix-starting SMILES input but with prefix control deactivated. This condition, referred to as the prefix₀ input, allowed the model to generate molecules freely without enforcing the fixed substructure. Additionally, models were tested using RDKit canonicalised SMILES inputs. For consistency, the Mol2Mol model was also evaluated under the prefix₀ input condition. To enable this, the internal SMILES standardisation procedure implemented in Mol2Mol was disabled, allowing the model to accept input SMILES in their original, user-defined form.

## Results and discussion

### Effect of SMILES alignment

To enhance the model’s ability to learn structural transformations between molecule pairs, SMILES alignment using Levenshtein distance was applied during preprocessing. Three datasets were constructed: Canonical (canonical SMILES pairs), Randomised (shuffled atom indices), and Levenshtein-aligned (Lev_aligned; minimal character-level differences). Separate Seq2Seq models were trained on each dataset for 100 epochs. Models were evaluated using greedy search, generating one SMILES per source input, with canonical and randomised SMILES inputs used to assess structural validity and similarity to source and target molecules.

**Fig. 3:**
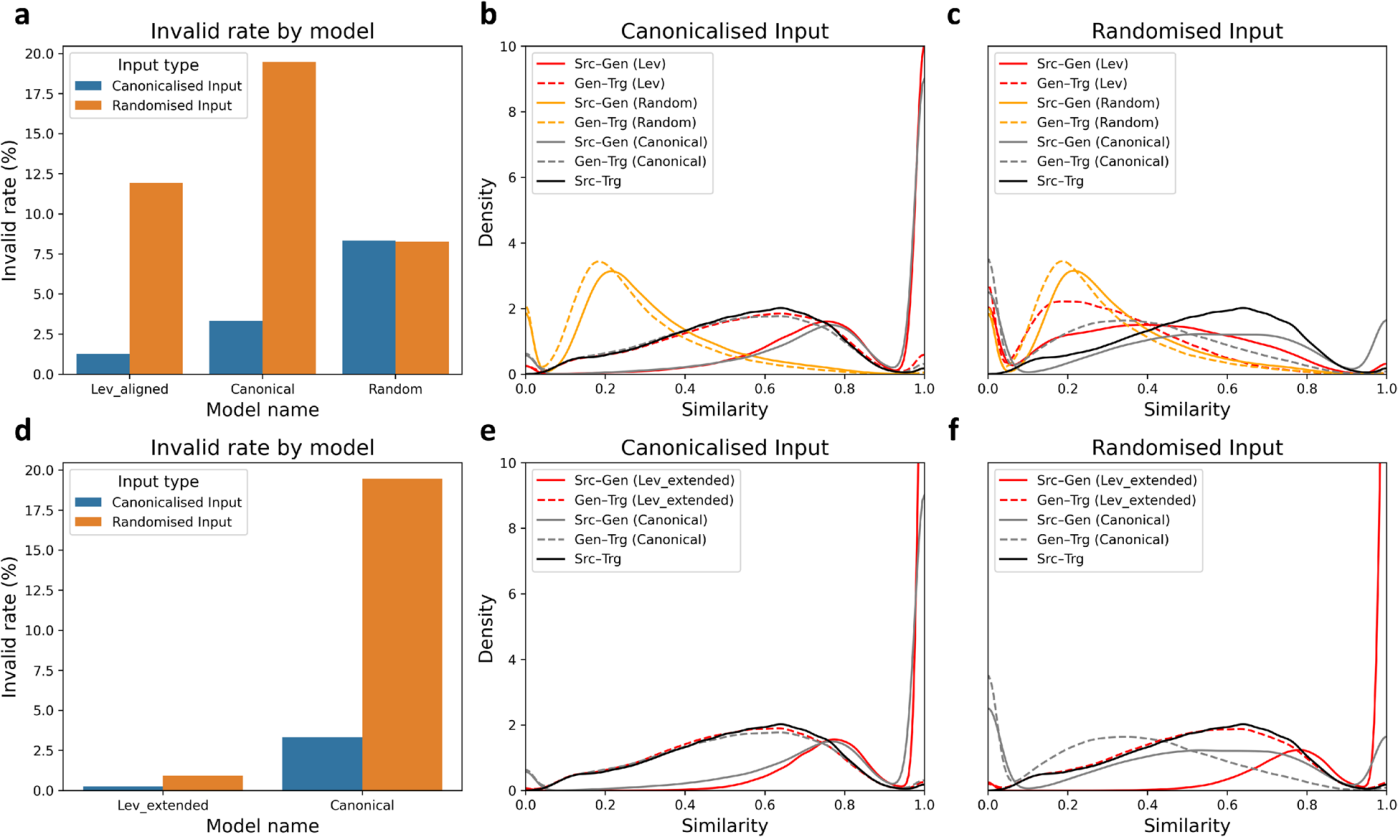
**SMILES alignment model evaluation. **Performance evaluation of SMILES-alignment models across two experimental stages: **a** invalid rates for models trained on Canonical, Random, and Lev_aligned SMILES datasets, evaluated using canonical (blue) and randomised (orange) input SMILES; **b, c** structural similarity distributions among source (Src), target (Trg), and generated (Gen) molecules produced by these models when evaluated with canonical (**b**) and randomised (**c**) inputs, with solid lines representing Src–Gen similarity, dotted lines indicating Gen–Trg similarity, and the solid black line showing the baseline Src–Trg similarity. Colours distinguish Canonical, Random, and Lev_aligned models; **d** invalid rates for the Lev_extended model compared to the Canonical model, evaluated with canonical (blue) and randomised (orange) inputs; **e, f** structural similarity distributions for Lev_extended and Canonical models evaluated with canonical (**e**) and randomised (**f**) input SMILES. Line styles retain previously defined meanings, and colours differentiate Lev_extended and Canonical models

When evaluated using canonicalised SMILES inputs, models trained on Canonical and Levenshtein-aligned (Lev_aligned) SMILES datasets showed strong generation validity (Fig. 3a). The Canonical model had an invalid rate of approximately 3%, whereas the Lev_aligned model exhibited an even lower invalid rate of around 1.3%. Structural similarity analyses further demonstrated that both models consistently generated molecules highly similar to their respective source structures, with more than 75% of the generated SMILES achieving Tanimoto similarity greater than 0.7 (Lev_aligned: 77.3%, Canonical: 79.3%; Fig. 3b). Additionally, similarity distributions comparing generated molecules with their corresponding target structures closely matched the original source-target distribution, confirming that structural features of the source molecules were well preserved.

However, model performance notably declined when tested with randomised SMILES inputs. Under these conditions, the Canonical model exhibited markedly reduced robustness, with the invalid rate increasing to approximately 20%. Although the Lev_aligned model was comparatively more robust, its invalid rate still increased substantially to around 11% (Fig. 3a). Structural similarity values generated by both the Canonical and Lev_aligned models under randomised input conditions varied broadly (approximately 0.2–0.7; Fig. 3c), demonstrating significant instability. This decline indicates that both models had limited capacity to interpret structurally equivalent but sequentially diverse SMILES representations, likely due to insufficient exposure to diverse structural representations during their training.

In contrast, the Random model, trained exclusively on SMILES pairs where both SMILES strings were randomised, consistently demonstrated poor performance irrespective of input format, maintaining an invalid rate around 8% (Fig. 3a). Structural similarity analyses further indicated that generated molecules consistently lacked structural relevance to either source or target structures, with similarity values remaining consistently low (approximately 0.2; Fig. 3b, c). This highlights that training solely on randomised SMILES severely limits the model’s ability to learn chemically meaningful transformations.

### Data augmentation with SMILES alignment

To address the limited robustness of the Lev_aligned models observed under randomised input conditions, the training dataset was augmented by incorporating additional randomised SMILES pairs, while still maintaining SMILES alignment to emphasise structurally relevant transformations. A new model, Lev_extended, was subsequently trained on this augmented dataset. This approach aimed to enhance model performance by exposing the model to diverse SMILES representations, enabling better generalisation across varied input formats.

The Lev_extended model, trained using the augmented dataset, was benchmarked against the previously established Canonical model (Fig. 3d). The Lev_extended model exhibited significantly lower invalid rates (canonicalised input: ~ 0.2%; randomised input: ~ 0.9%), demonstrating improved robustness across different input formats. This improvement arises primarily from two factors: first, data augmentation provided diverse SMILES representations of the same structural transformations within molecule pairs during training, facilitating better generalisation across varied input representations; second, under greedy search decoding method, the model frequently returned identical SMILES to the input molecule, as the input inherently represents maximum structural similarity.

Structural similarity analyses (Fig. 3e, f) further supported these improvements. Regardless of input format, the majority of generated molecules were identical to their corresponding source structures, with identity rates exceeding 60% for both canonicalised (63.1%) and randomised inputs (69.0%). Even non-identical generated molecules consistently showed high structural similarity (~ 0.8), indicating the model reliably produced molecules closely correlated to the input.

In summary, data augmentation combined with SMILES alignment significantly enhanced model robustness and enabled reliable generation of structurally correlated molecules. Therefore, the Lev_extended model was chosen for SMILES generation in ANNalog.

### Structural properties of generated molecules

An independent test set of 1000 ChEMBL35 assays, non-overlapping with training data, was selected to evaluate ANNalog. For each assay, ANNalog generated between 50 and 1000 SMILES per input molecule using beam search and multinomial sampling methods. The analyses conducted aimed to assess structural similarity to assay targets (the molecules within each assay exhibiting the highest structural similarity, defined as the global target), correlate generation probabilities with structural similarity, and profile the diversity of functional groups and ring systems to investigate the structural properties of generated molecules.

**Fig. 4:**
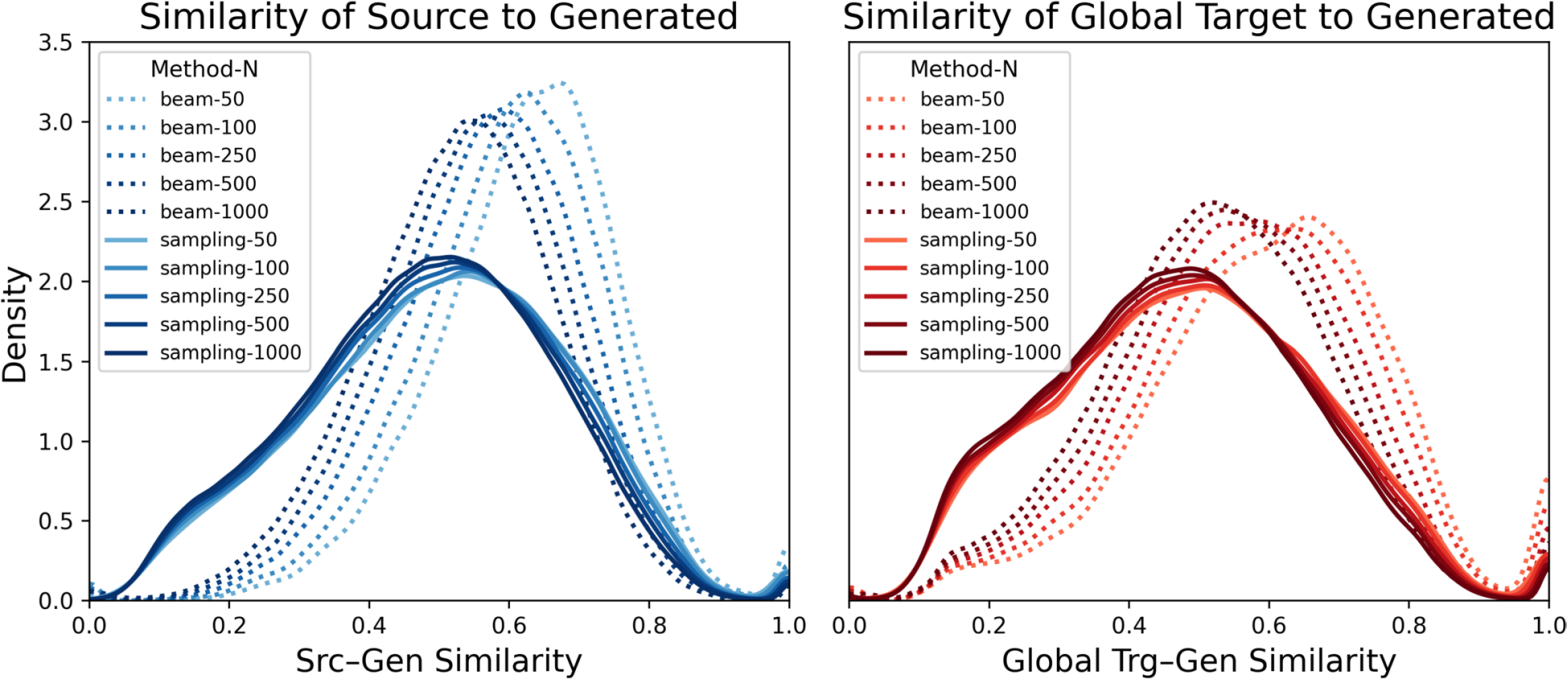
**Similarity distribution analysis.** Similarity distributions comparing generated SMILES to the source and global target molecules. Beam search (beam) method is represented by dotted lines, and the multinomial sampling (sampling) method by solid lines. Colour intensity indicates the number of generated SMILES (N = 50, 100, 250, 500, 1000) per set, with darker shades corresponding to larger generated sets

Structural similarity distributions comparing generated SMILES to both source and global target molecules are illustrated in Fig. 4. Overall, SMILES generated using the beam search method exhibited notably higher structural similarity to the source molecules compared to those generated via the multinomial sampling method, particularly when fewer SMILES were generated. For instance, among 50 SMILES generated by beam search, half showed similarity scores above 0.68 to the source inputs, with more than 30% surpassing 0.75. In contrast, only 9% of the SMILES produced by the multinomial sampling method exceeded the 0.75 similarity threshold. When comparing generated molecules with global targets, a similar trend was observed: 50% of the SMILES from beam search had similarity scores greater than 0.66, with approximately 30% surpassing 0.75. By comparison, multinomial sampling yielded markedly lower similarities, with median similarity scores around 0.5 and only 11% of generated SMILES surpassing the 0.75 threshold. Furthermore, with beam search, the fraction of outputs identical to the global target was 5.0% when generating 50 SMILES and 2.8% when generating 100 SMILES. This suggests that, for this task and decoding setup, generating 50 SMILES already provides near saturated target recovery, and increasing the sample size yields limited additional benefit for recovering the global target.

The results from Fig. 4 indicate that the ANNalog model is effective at generating structurally similar analogues. The beam search method demonstrates superior performance, as evidenced by its capacity to preferentially generate molecules that closely resemble the source molecules. This aligns with the inherent logic of beam search, which prioritises molecules based on model-assigned probabilities, initially selecting structures with higher probabilities that typically exhibit higher structural similarity. This indicates that molecules assigned higher generation probabilities by the model closely align with chemical features present in the input molecules and relevant assay targets. As the beam width increases, molecules with progressively lower probabilities and correspondingly lower similarity are generated. This correlation is further validated by Supplementary Figure S3, where the heatmap clearly illustrates that molecules with higher model-assigned generation probabilities consistently show greater similarity to their respective source molecules.

In contrast, the multinomial sampling method directly explores a broader chemical space, often generating structures with substantially lower similarity scores relative to the source molecules. This broader search capability is beneficial for discovering structurally diverse analogues, particularly regarding the diversity of newly introduced functional groups and ring systems. The structural diversity introduced by the multinomial sampling method scales proportionally with the size of generated SMILES sets, as revealed by Supplementary Figure S4 and detailed in Supplementary Figure S5. The multinomial sampling method frequently produced modifications involving halogen substitutions (Cl, Br, F), the introduction of alkoxy (–OR) groups, hydroxyl (–OH) functionalities, and significant alterations to ring systems, particularly the replacement of ring carbon atoms with heteroatoms such as nitrogen, oxygen, and sulfur.

In summary, both methods, beam search and multinomial sampling, demonstrate their effectiveness in generating analogues similar to the query molecules. Beam search excels at initially identifying molecules structurally similar to the source, with similarity declining as the search expands. This property can strategically be exploited: by requesting larger beam searches, researchers can progressively widen the chemical search space when initial results are insufficient. On the other hand, multinomial sampling inherently provides rapid exploration of a more structurally diverse chemical landscape, beneficial for identifying novel modifications to functional groups and ring systems.

## Ability to scaffold hop

### Generation of neighbours around MedChem-similar targets

From the previously used 1000 ChEMBL35 assays, 108 molecule pairs were manually selected, representing scaffold and ring-system transformations (the source target similarity distribution of these 108 pairs is provided in the Supplementary Figure S6). ANNalog and an external model Mol2Mol were compared by performing two sets of analyses: evaluation of model generation probabilities indicating the likelihood of generating target molecules from source inputs, and evaluation of structural similarity to targets.

Negative log-likelihood values for generating the exact target from the source were consistently lower for ANNalog than for Mol2Mol [16] across canonicalised, randomised, and aligned SMILES representations, as detailed in Supplementary Figure S7. This indicates that ANNalog assigns higher likelihood to the curated target structures in this benchmark. This trend is consistent with the different intended use of the two models, as Mol2Mol is designed for local analogue generation around an input structure and is therefore expected to favour near neighbour transformations, which can reduce the likelihood assigned to targets requiring larger scaffold or ring system changes.

**Fig. 5:**
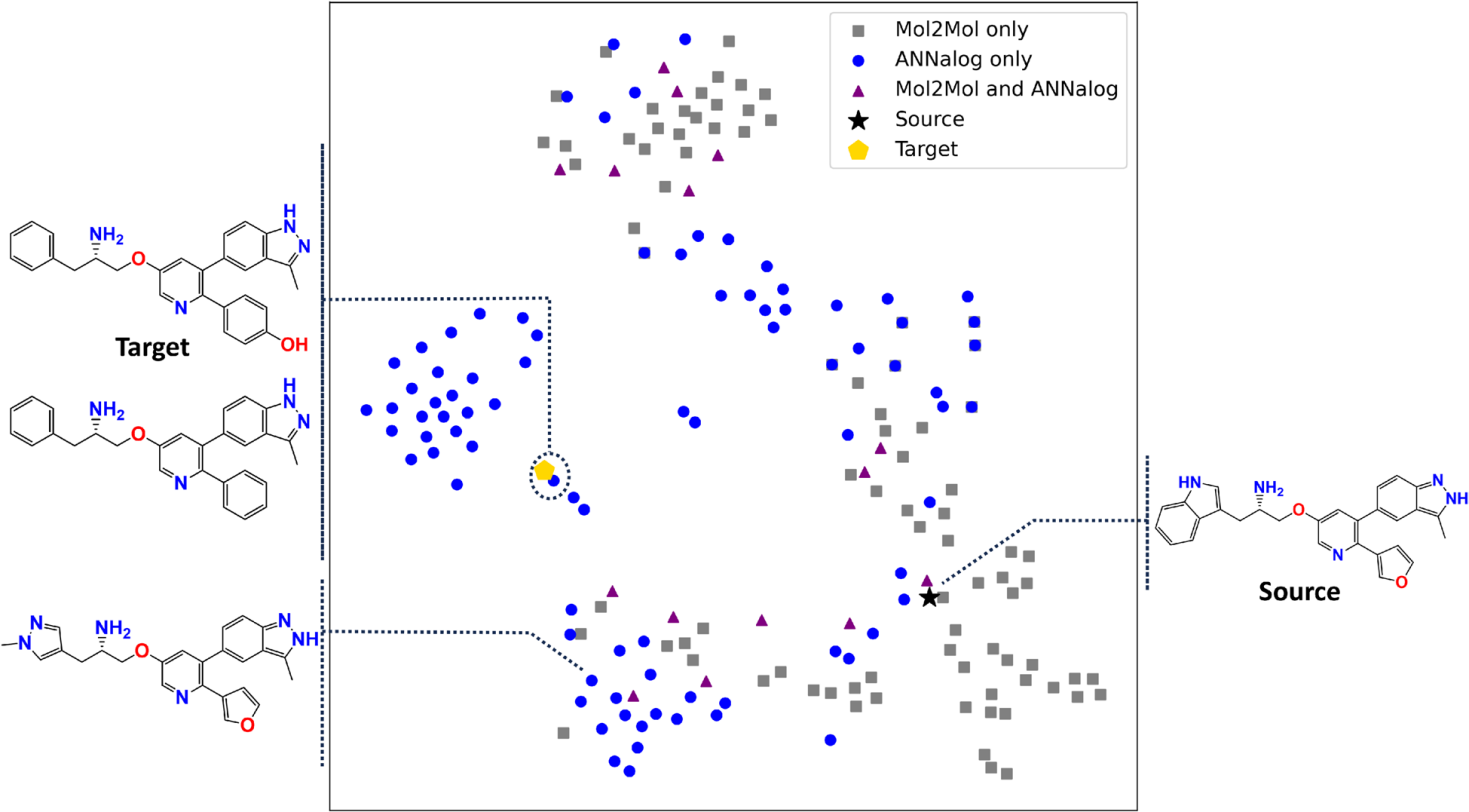
**t-SNE embedding of generated molecules.** t-SNE projection of ANNalog and Mol2Mol generated molecules for one source–target pair, based on Morgan fingerprints and Jaccard distance. The target is shown as a yellow pentagon, the source as a black star, ANNalog-generated molecules as blue circles, Mol2Mol-generated molecules as grey squares, and molecules generated independently by both models (Mol2Mol and ANNalog) are shown as purple triangles. Highlighted structures include the ANNalog-generated molecule closest to the target, which closely recovers the scaffold transformation, and an additional ANNalog-generated molecule from a more distant region of the embedding space, illustrating alternative scaffold modifications

Figure 5 shows an example of one source–target molecular pair, visualised alongside the two-dimensional t-distributed stochastic neighbour embedding projection of molecules generated by both ANNalog and Mol2Mol. For this illustrative example, each model generated 100 unique molecules from the source molecule using the beam search method. The Mol2Mol model primarily generated molecules located close to the input molecule in chemical space, which is consistent with its intended use for local chemical-space exploration, and its generated neighbours remained highly similar to the source, spanning Tanimoto similarities between 0.7241 and 1.0, calculated with bit-based ECFP4 fingerprints (16,384 bits). While ANNalog also produced molecules within this same region, it additionally generated molecules that project to other regions of the two-dimensional embedding that were not populated by Mol2Mol in this example, suggesting a broader range of structural modifications under the same generation number. This broader spread is also reflected in the similarity range, which spans 0.4699 to 1.0 and extends down to the source–target similarity for this pair, 0.4713, whereas the Mol2Mol model does not reach this similarity level under the same generation number. Additional examples in which Mol2Mol generates structures closer to the target than ANNalog are provided in Supplementary Figure S8.

In terms of exploring the chemical space around the target, ANNalog demonstrates broader generative capacity compared to Mol2Mol. In the example shown in Fig. 5, Mol2Mol primarily generated close analogues that remain strongly related to the source structure. By contrast, the nearest neighbour to the target is highlighted, showing a molecule generated by ANNalog that not only recovers the key scaffold transformation from indole (in the source) to benzene (in the target), but also extends this transformation by replacing the furan ring with a benzene, missing only the hydroxyl group to fully reconstruct the target. This illustrates the model’s ability to approach the target structure through substantial scaffold edits. In addition, another ANNalog-generated molecule from a more distant cluster is highlighted, which does not converge on the target scaffold but instead reflects alternative scaffold modifications.

**Fig. 6:**
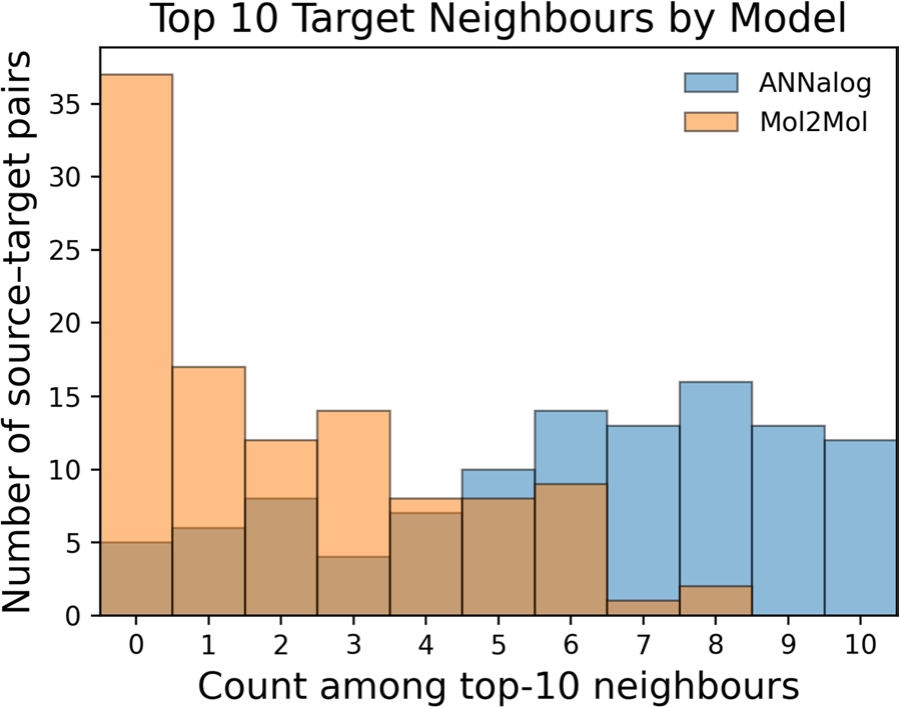
**Model origin of the ten target-nearest generated molecules.**For each source–target pair, ANNalog and Mol2Mol outputs were pooled, ranked by structural similarity to the target, and the ten closest generated molecules were identified. The histograms show how many of these ten were contributed by ANNalog (blue) or Mol2Mol (orange) across 108 pairs.

To demonstrate that the capability illustrated above is representative rather than isolated, the origin of the top ten generated molecules among all generated ones exhibiting the highest structural similarity to each target molecule was analysed across all 108 source–target pairs and summarised as overlapping histograms in Fig. 6. In more than 50% of the analysed cases, over half of the top ten nearest neighbours originated from ANNalog, and ANNalog exclusively generated all ten nearest neighbours for 12 of the 108 pairs. By comparison, in approximately 34% of the pairs, none of the top ten nearest neighbours originated from Mol2Mol under the same generation number. This observation is consistent with the different generative tendencies of the two models. Across the 108 inputs, Mol2Mol largely follows its intended use of enumerating close analogues around an input structure, producing outputs that remain strongly similar to the source and typically fall in the 0.6 to 1.0 similarity range to the input, whereas the curated source–target pairs in this experiment have lower source–target similarity, predominantly around 0.4 to 0.5. The relationship between the target similarity and the similarity neighbourhood sampled by each model is illustrated in more detail in Supplementary Figure S9. Overall, these results further support ANNalog’s ability to generate relevant molecular analogues and recover meaningful scaffold modifications when target structures differ substantially from their corresponding source inputs.

### Scaffold hopping with prefix control

**Fig. 7:**
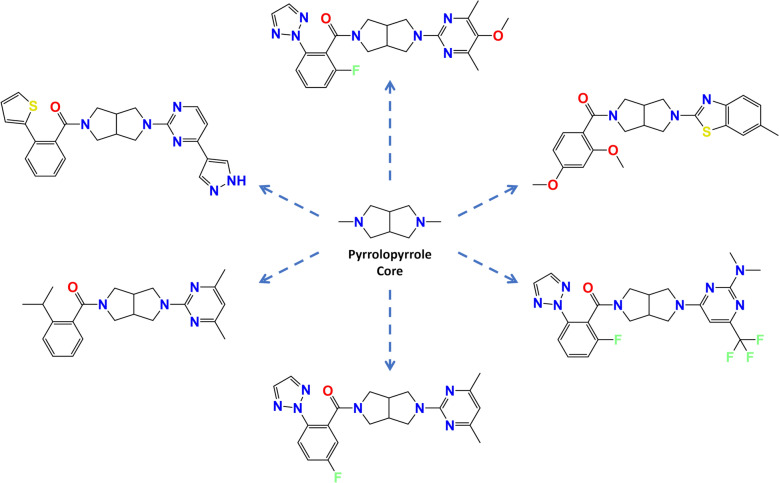
**Pyrrolopyrrole core and representative antagonists.** The octahydropyrrolo[3,4-c]pyrrole core structure (centre), surrounded by six OX2 receptor antagonist structures from the 380 exemplified in the patent for illustration

**Fig. 8:**
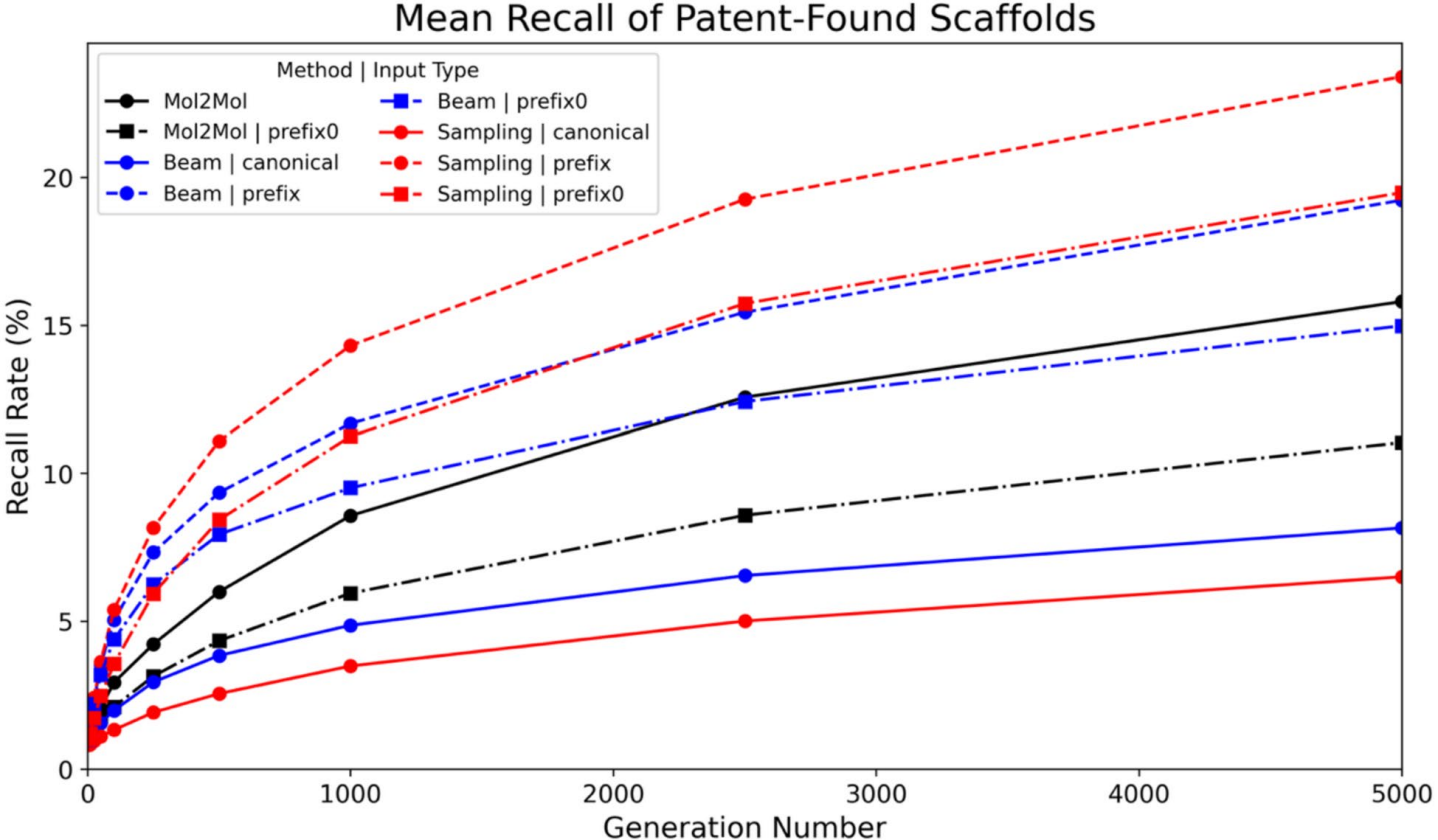
**Scaffold recall analysis. **Mean scaffold recall rate (%) on the orexin-2 antagonist dataset as a function of generation number (number of SMILES generated per input), averaged over 380 inputs (up to 5000 SMILES per input). ANNalog results are shown for beam search (blue) and multinomial sampling (red), and Mol2Mol results are shown for beam search (black). Solid lines denote canonicalised inputs, dashed lines with circle markers denote prefixed inputs with prefix control enabled, and dash-dot lines with square markers denote prefix₀ inputs without prefix control

In this part of the experiment, scaffold hopping ability was assessed using 380 octahydropyrrolo[3,4-c]pyrrole-based orexin-2 receptor (OX2) antagonists exemplified in the patent (US11059828B2) [35], covering 121 unique Bemis-Murcko scaffolds. Representative structures are shown in Fig. 7. Three input representations were considered: RDKit canonicalised SMILES, prefix-controlled inputs that fix the octahydropyrrolo[3,4-c]pyrrole substructure at the start of the SMILES during generation, and prefix₀ inputs that place the same core substructure at the start of the input SMILES without enforcing it during generation. Molecules were generated using either beam search or multinomial sampling. Each evaluated setting was run once using a single input SMILES per molecule. Beam search decoding is deterministic for a fixed model and input representation, whereas multinomial sampling was also evaluated using a single run per setting. For each evaluated combination of model, input representation, and decoding method, all 380 exemplified molecules were used as inputs and up to 5000 SMILES were generated per input molecule. Prefix controlled inputs were evaluated for ANNalog only, as enforcing an equivalent prefix constraint for Mol2Mol would require source code level modifications. Model performance was quantified using scaffold recall, defined per input as the proportion of unique Bemis-Murcko scaffolds recovered from valid generated molecules relative to the set of unique patent scaffolds excluding the scaffold of the input molecule. Figure 8 reports mean recall rates obtained by averaging the per-input recall across the 380 inputs.

The scaffold recall rates achieved by the ANNalog and Mol2Mol models on the orexin-2 antagonist dataset, across varying numbers of generated molecules per input, are presented in Fig. 8. When RDKit canonicalised SMILES were used as input, Mol2Mol achieved a scaffold recall rate around 15% using beam search, whereas ANNalog reached approximately 8% with beam search and around 5% with multinomial sampling under the same input condition. Notably, although Mol2Mol was not originally designed for scaffold hopping, under the large generation number used here it still recovered a substantial fraction of patent scaffolds distinct from the input scaffold.

In contrast, the application of prefix control, specifically fixing the octahydropyrrolo[3,4-c]pyrrole core at the beginning of the input SMILES, resulted in substantial performance gains for ANNalog. Under prefix controlled conditions, ANNalog achieved a scaffold recall rate approaching 25% with the multinomial sampling method and slightly below 20% with the beam search method. Under these prefix controlled settings, ANNalog achieved higher scaffold recall than Mol2Mol under RDKit canonicalised inputs.

To facilitate comparison under a similar input formatting, a prefix₀ condition was evaluated, where prefix-formatted SMILES were used as input without enforcing the prefix during generation. For Mol2Mol, this was enabled by turning off input standardisation so that the model could accept the same prefix₀ formatted inputs. Under this setting, ANNalog achieved scaffold recall rates of approximately 20% using multinomial sampling and 15% using beam search, while Mol2Mol achieved approximately 10%. Removing prefix control reduced ANNalog performance relative to the prefix controlled setting, but ANNalog remained higher than Mol2Mol under the prefix₀ input condition.

The improved recall observed with prefix₀ input compared to canonical input may reflect ANNalog’s exposure to aligned SMILES pairs during training, which often share common prefixes, together with the left-to-right decoding bias of autoregressive generation that tends to preserve early tokens and introduce edits later in the sequence. This training and decoding behaviour could encourage preservation of initial SMILES tokens during generation. In this experiment, placing the core pyrrolo[3,4-c]pyrrole structure at the beginning of the input SMILES may therefore increase the likelihood of retaining this motif in generated scaffolds. By comparison, Mol2Mol performed worse under the prefix₀ input condition than with canonical SMILES, which is consistent with its training on canonicalised representations rather than randomised or aligned SMILES variants.

The ~ 25% scaffold recall rate achieved under prefix control leaves clear room for improvement. In this study, multiple alternative prefix encodings that represent the same input partial structure were not evaluated. In practical use, generation can be performed from several SMILES representations of the same molecule that all satisfy the desired prefix control, which may increase the probability of sampling additional scaffolds and thereby improve recall. Moreover, subject to available computational resources, increasing the generation number is a straightforward option. As indicated in Fig. 8, none of the evaluated models show a clear scaffold recall plateau within the tested range, suggesting that additional sampling is likely to further increase recall.

These results support the use of ANNalog for scaffold hopping tasks where control over the direction of scaffold modification is desirable. Placing a relevant substructure at the beginning of the input SMILES and enabling prefix control can guide generation towards retaining key structural motifs while exploring scaffold variations. When greater flexibility is desired, using the same prefixed input format without enforcing prefix control provides an alternative setting that still biases generation towards the initial substructure while allowing a broader range of scaffold modifications.

## Conclusion

We introduce ANNalog, a transformer-based sequence-to-sequence generative model specifically developed to produce MedChem-similar molecular analogues. ANNalog is trained on molecule pairs extracted from ChEMBL33, based on the rationale that molecules tested within the same biological assay represent chemically relevant analogues. The dataset, composed of molecule pairs derived from approximately 31,000 assays, was pre-processed through a SMILES alignment technique based on Levenshtein distance, highlighting structural transformations between paired molecules and enhancing the model’s focus on chemically meaningful differences. Further performance improvements were obtained by augmenting the training data with additional aligned SMILES pairs generated via atom-index randomisation.

ANNalog predominantly generates molecules structurally similar to input ligands, frequently involving subtle structural modifications such as substituent replacements or additions. Additionally, scaffold hopping analyses showed ANNalog’s capability to generate structurally diverse yet medicinally relevant scaffolds. Specifically, when evaluated against manually curated molecular pairs with known scaffold hops, ANNalog consistently generated analogues structurally neighbouring the intended target scaffolds.

In a case study involving orexin-2 receptor antagonists derived from patent literature, we employed ANNalog’s built-in prefix control mechanism, which allows specific structural motifs within input molecules to be fixed. Under these constrained scaffold hopping conditions, ANNalog successfully rediscovered known patent scaffolds, achieving a scaffold recall rate of approximately 25%, underscoring its capability for guided scaffold exploration.

In summary, ANNalog effectively balances the generation of structurally similar analogues and scaffold hopping towards medicinally relevant chemical spaces. Importantly, scaffold hopping directions can be systematically controlled via prefix-guided generation, making ANNalog a valuable and practical tool for molecular optimisation tasks in AI-driven drug discovery programmes.

In future work, for data augmentation, beyond simply enlarging the dataset, we could try to explore a two-stage training strategy in which a model is first trained on a canonicalised and aligned dataset and then fine-tuned on a randomised, aligned dataset, to assess whether this improves how efficiently the model learns to handle canonicalised SMILES. We also plan to extend our evaluation to a deeper iterative setting, where generated molecules are fed back as inputs for subsequent rounds of generation, enabling a more thorough assessment of generative quality. Finally, given Mol2Mol’s focus on the enumeration of near neighbours around an input molecule, a promising direction is to combine the two models, for example by using ANNalog for scaffold hopping and then applying Mol2Mol to perform neighbourhood searching around ANNalog-generated candidates.

## Supplementary Information

**Additional file 1**.

## Data Availability

The code and implementation details for the ANNalog model described in this study are publicly available at: https://github.com/DVNecromancer/ANNalog/tree/main. The training dataset is available at 10.5281/zenodo.17148777. Additionally, an online version of ANNalog is accessible via Google Colab at: https://colab.research.google.com/drive/1aJhaBOG7xuYFwMGzfUmbMsLe8T462Ptc#scrollTo=J_uXqGbqK-Ol

## Abbreviations

VAE: Variational autoencoder
RNN: Recurrent neural networks
Seq2Seq: Sequence-to-sequence
OX2: Orexin-2 receptor
SMILES: Simplified molecular input line entry system
