# Supplementary material for: ANNalog: generation of MedChem-similar molecules
Source: J Cheminform. 2026 Apr 15;18:53. doi: 10.1186/s13321-026-01186-6 (PMC13126752; doi:10.1186/s13321-026-01186-6)
Supplement: Supplementary file 1 — Additional file 1. [file 13321_2026_1186_MOESM1_ESM.docx]

**ANNalog — Generation of MedChem-similar Molecules**

Wei Dai^1, 2^*, Jonathan D Tyzack^2^, Arianna Fornili^1^, Chris de Graaf^3^, Noel M O’Boyle^4^*

1. School of Physical and Chemical Sciences, Queen Mary University of London, E1 4NS, UK
2. Nxera Pharma, Steinmetz Building, Granta Park, Great Abington, Cambridge, CB21 6DG, UK
3. Structure Therapeutics, 601 Gateway Blvd, Suite 900, South San Francisco, CA 94080, USA
4. EMBL’s European Bioinformatics Institute (EMBL-EBI), Wellcome Genome Campus, Hinxton, Cambridgeshire, CB10 1SD, UK

*Correspondence:
Noel M O’Boyle: [oboyle@ebi.ac.uk](mailto:oboyle@ebi.ac.uk)
Wei Dai: w.d.dai@qmul.ac.uk

**Supplementary Information**

**Table T1.** Selected source–target molecule pairs with scaffold transformations used for generative model benchmarking.

| **Document ID** | **Assay ID** | **Source molecule SMILES** | **Target molecule SMILES** |
| --- | --- | --- | --- |
| **CHEMBL1130772** | **CHEMBL646840** | **C[C@H]1CC[C@H]2Nc3cc4c(cc3[C@H]2C1)c(C(F)(F)F)cc(=O)n4C** | **CN1CCc2cc3c(C(F)(F)F)cc(=O)n(C)c3cc21** |
| **CHEMBL1149152** | **CHEMBL677037** | **Cc1cccc(COc2nn3c(-c4ccccc4)nnc3c3c2C2CCC3CC2)n1** | **Cc1cccc(COc2nn3c(-c4cc(C)on4)nnc3c3ccccc23)n1** |
| **CHEMBL4616703** | **CHEMBL4619017** | **CN1C[C@H](Cc2ccc(Cl)cc2)N(C2CCN(c3n[nH]c(N)n3)CC2)Cc2ccccc21** | **Nc1nc(N2CCC(N(CCc3ccc(Cl)cc3)Cc3cccc(C(F)(F)F)c3)CC2)n[nH]1** |
| **CHEMBL2069280** | **CHEMBL2073113** | **CCc1nc(-c2ccc(S(C)(=O)=O)c([N+](=O)[O-])c2)sc1C(=O)N1CC2(C)CC1CC(C)(C)C2** | **CC1CCN(C(=O)c2sc(-c3ccc(S(C)(=O)=O)c([N+](=O)[O-])c3)nc2C(F)(F)F)CC1** |
| **CHEMBL2375281** | **CHEMBL2378591** | **O=C(NC1(C(=O)NO)CCNCC1)c1ccc(C#Cc2ccccc2)cc1** | **CC(C)(NC(=O)c1ccc(C#Cc2ccccc2)cc1)C(=O)NO** |
| **CHEMBL4145565** | **CHEMBL4149394** | **N#Cc1cc2c3c(c1)c(O)c(C(=O)NCC(=O)O)c(=O)n3CC(c1ccc(C(F)(F)F)cc1)O2** | **O=C(O)CNC(=O)c1c(O)c2c(n(Cc3ccc(C(F)(F)F)cc3)c1=O)CCNC2** |
| **CHEMBL2177091** | **CHEMBL2185270** | **C[C@H]1C[C@]2(CCN1Cc1cccc(-c3ccccc3Cl)c1)CNS(=O)(=O)N2c1cccc(F)c1** | **CC(C)Oc1cc(CN2CC[C@@]3(C[C@@H]2C)CN(C)S(=O)(=O)N3c2cccc(F)c2)ccc1O** |
| **CHEMBL1136537** | **CHEMBL652141** | **N#Cc1c(N2CC3CCC(CC3)C2)[nH]c2cccnc12** | **CN(C)CCN(C)c1[nH]c2cccnc2c1C#N** |
| **CHEMBL1153241** | **CHEMBL1071954** | **Cc1[nH]nc2ccc(-c3cc(OC[C@@H](N)Cc4c[nH]c5ccccc45)cnc3-c3ccoc3)cc12** | **Cc1n[nH]c2ccc(-c3cc(OC[C@@H](N)Cc4ccccc4)cnc3-c3ccc(O)cc3)cc12** |
| **CHEMBL5113400** | **CHEMBL5115327** | **CCN1CCN(c2cc(NC(=O)[C@@H]3C[C@H](n4cc(-c5ccc6c(N)n[nH]c6c5)nn4)CN3)ccc2Cl)CC1** | **O=C(NC1CC1)c1cncc(-c2cn([C@@H]3CN[C@H](C(=O)Nc4ccc(Cl)c(C(F)(F)F)c4)C3)nn2)c1** |
| **CHEMBL5329201** | **CHEMBL5330459** | **CCNC(=O)Nc1cc(-c2cccnc2)cc2c(C(C)=O)c(C)[nH]c12** | **CCOC(=O)Nc1cc(C(=O)OC)cc2c(C(C)=O)c(C)[nH]c12** |
| **CHEMBL1135749** | **CHEMBL758927** | **O=c1[nH]cnc2c1ncn2[C@@H]1S[C@H](CO)C=C1F** | **Cc1cn([C@@H]2S[C@H](CO)C=C2F)c(=O)nc1O** |
| **CHEMBL1132611** | **CHEMBL763668** | **COc1cc2cc(CO)cc(-c3ccnc(-n4nc(-c5cccnc5)c5ccccc5c4=O)c3)c2cc1OC** | **COc1cc2cc(CO)c(CO)c(-c3ccnc(-n4c(=O)ccc5ccccc54)c3)c2cc1OC** |
| **CHEMBL1133950** | **CHEMBL643554** | **Nc1ncnc(N[C@@H]2O[C@H](CO)[C@@H](O)[C@H]2O)c1C1=NCCO1** | **Nc1ncnc(N[C@@H]2O[C@H](CBr)[C@@H](O)[C@H]2O)c1[N+](=O)[O-]** |
| **CHEMBL3727346** | **CHEMBL3734188** | **Cn1c(N2CCN[C@@H](c3ccc(-c4ncon4)cc3)C2)nc(-c2ccncc2F)cc1=O** | **CN(C)CC(=O)Nc1ccc([C@H]2CN(c3nc(-c4ccncc4F)cc(=O)n3C)CCO2)cc1** |
| **CHEMBL1155106** | **CHEMBL990108** | **O=C1c2ccccc2C2(CCCC2)C(=O)N1c1ccc(Cl)cc1** | **COc1ccc(N2C(=O)c3ccccc3C(C)(C)C2=O)cc1** |
| **CHEMBL1128174** | **CHEMBL785004** | **OCCN1CCN(C2CC(c3ccc(F)cc3)c3ccc(Cl)cc32)CC12CCCC2** | **CN1CCN(C2CC(c3ccc(F)cc3)c3ccc(F)cc32)CC1** |
| **CHEMBL1133879** | **CHEMBL662153** | **O=C(Nc1ccc(F)cc1)c1ccc(S(=O)(=O)Cc2ccccc2)[n+]([O-])c1** | **O=C(NCc1ccc(F)cc1)c1ccc(Cl)[n+]([O-])c1** |
| **CHEMBL1240529** | **CHEMBL1246850** | **CS(=O)(=O)c1ccc(C(=O)c2ccn(-c3ccccc3F)c2)cc1** | **CS(=O)(=O)c1ccc(C(=O)c2ccoc2)cc1** |
| **CHEMBL1144028** | **CHEMBL950176** | **Cc1ccc(NC(=O)N2CCOc3cc(Oc4ncnc5[nH]ccc45)ccc32)cc1** | **COc1cc2nccc(Oc3ccc4c(c3)OCCN4C(=O)NC(C)C)c2cc1OC** |
| **CHEMBL3751732** | **CHEMBL3755806** | **Cc1ccc(CNc2nc3c(c(N4CCN(C)CC4)n2)CCc2ccccc2-3)cc1** | **CN1CCN(c2cc(-c3ccccc3)nc(NCc3ccccc3)n2)CC1** |
| **CHEMBL1139329** | **CHEMBL1018742** | **Cc1oc(S(=O)(=O)N2CCCCC2)cc1NC(=O)N[C@@H](C)c1ccccc1** | **Cc1oc(S(=O)(=O)N(C)C)cc1NC(=O)Nc1ccccc1** |
| **CHEMBL3588817** | **CHEMBL3592256** | **Nc1nc2c(s1)C[C@@H](NC(=O)c1cc3cc(Cl)ccc3[nH]1)CC2** | **COC(=O)CC(=O)Nc1nc2c(s1)C[C@@H](NC(=O)c1cc(Br)c(Br)[nH]1)CC2** |
| **CHEMBL1145296** | **CHEMBL908461** | **CC(=O)N1CCN(Cc2ccc3[nH]c(-c4cc5cc(-c6cn[nH]c6)ccc5[nH]c4=O)cc3c2)CC1** | **NCC1CCN(Cc2ccc3[nH]c(-c4cc5cc(C(N)=O)ccc5[nH]c4=O)cc3c2)CC1** |
| **CHEMBL1154183** | **CHEMBL1046767** | **COc1ccccc1-n1c(-c2cc(O)ccc2C)cn2c3c(=O)[nH]c(=O)n(C)c3nc12** | **Cn1c(=O)[nH]c(=O)c2c1nc1n(CCCCO)c(-c3ccccc3)cn21** |
| **CHEMBL3872259** | **CHEMBL3874742** | **c1ccc(-c2n(Cc3ccc4ccccc4n3)cc[n+]2Cc2ccc3ccccc3n2)cc1** | **CCCCc1n(Cc2ccc3ccccc3n2)cc[n+]1Cc1ccc2ccccc2c1** |
| **CHEMBL1146681** | **CHEMBL818934** | **CS(=O)(=O)N1CCN(Cc2ccc(-c3ccn4c(-c5ccccc5)cnc4c3)cc2)CC1** | **O=c1cc(-c2ccn3c(-c4ccccc4)cnc3c2)ccn1CCCO** |
| **CHEMBL3351756** | **CHEMBL3390204** | **O=C1c2ccccc2-c2c1c(N1CCN(c3ccccn3)CC1)nc1ccc(Cl)cc21** | **O=C1c2ccccc2-c2c1c(NCCN1CCOCC1)nc1cc(Cl)ccc21** |
| **CHEMBL4118268** | **CHEMBL4125215** | **O=S(=O)(NCc1ccccc1)c1cccc(Nc2ccc3nccc(-c4ccncc4)c3n2)c1** | **O=S(=O)(NCCO)c1cccc(-c2ccc3nccc(-c4ccc(OC(F)(F)F)cc4)c3n2)c1** |
| **CHEMBL4177539** | **CHEMBL4178356** | **C=CC(=O)N[C@@H]1CCN(c2nc(Nc3ccc(N4CCN(C)CC4)cc3)c3ncn(C(C)C)c3n2)C1** | **C=CC(=O)N[C@@H]1CN(c2nc(Nc3cn(C)nc3OC)c3ncn(C(C)C)c3n2)C[C@H]1F** |
| **CHEMBL2146434** | **CHEMBL2149358** | **Cc1c2c3cc(NC(=O)[C@@H]4CCCN4C(=O)CN)ccc3nc-2n(C)c2ccccc12** | **Cc1c2ccccc2nc2c1c1cc(NC(=O)CN)ccc1n2C** |
| **CHEMBL1151918** | **CHEMBL1034549** | **N=C(NC=C(c1ccccc1)c1ccccc1)NCCCc1c[nH]cn1** | **N=C(NCCc1ccc(Br)cc1)SCCCc1c[nH]cn1** |
| **CHEMBL2062411** | **CHEMBL2067159** | **N#CC1=C(N)OC2=C(C(=O)CN(Cc3ccccc3)C2)C1c1ccccc1** | **CC1C(C#N)=C(N)OC2=C1C(=O)CC(c1ccccc1)C2** |
| **CHEMBL3745705** | **CHEMBL3749262** | **C[C@H]1Cc2c(ncnc2Oc2ccc3c(ccn3C(=O)Nc3cc(C4(C)CC4)[nH]n3)c2)CN1** | **Cc1cncc(NC(=O)n2ccc3cc(Oc4ncnc5c4CCNC5)ccc32)c1** |
| **CHEMBL5042488** | **CHEMBL5043376** | **Cc1cc(C)cc(CNCC[C@@]2(c3ccccn3)CCOC3(CCOC3)C2)c1** | **FC1(F)CCc2csc(CNCC[C@@]3(c4ccccn4)CCOC4(CCCC4)C3)c2C1** |
| **CHEMBL4002609** | **CHEMBL4003960** | **Cc1ccc(-c2ccc(C(=O)N[C@H](C(=O)NO)C(C)(C)NS(C)(=O)=O)nc2)cc1** | **CC(C)(NS(C)(=O)=O)[C@H](NC(=O)c1ccc(-c2cc3ccccn3n2)cc1)C(=O)NO** |
| **CHEMBL1153282** | **CHEMBL960144** | **CN(C)c1ccnc(Nc2ccc(-c3nc4ccccc4s3)cc2)n1** | **COc1nc(Nc2ccc(-c3nc4ccccc4s3)cc2)c2cc[nH]c2n1** |
| **CHEMBL1144828** | **CHEMBL924741** | **O=c1on(CCCO)c(-c2ccncc2)c1-c1ccc(F)cc1** | **Cc1ccc(S(=O)(=O)n2oc(=O)c(-c3ccc(F)cc3)c2-c2ccncc2)cc1** |
| **CHEMBL1147483** | **CHEMBL733332** | **Fc1ccc(-c2nc(C3CCNCC3)cn2-c2ccnc(NC3CCCCC3)n2)cc1** | **CN1CCN(c2ccc3c(n2)nc(-c2ccc(F)cc2)n3-c2ccnc(NC3CCCC3)n2)CC1** |
| **CHEMBL4270594** | **CHEMBL4274107** | **O=c1[nH]c2ccccc2cc1-c1nc2cc(Cl)ccc2[nH]1** | **O=c1[nH]c2cc3c(cc2cc1-c1nc2cc(Br)ccc2[nH]1)OCO3** |
| **CHEMBL1914307** | **CHEMBL1921170** | **NC(=O)N1CCC(NC(=O)C(=O)Nc2ccc(Cl)cc2)CC1** | **O=C(Nc1ccc(Cl)cc1)C(=O)NC1CC2(CCCCC2)NC2(CCCCC2)C1** |
| **CHEMBL3880395** | **CHEMBL3880716** | **Cc1cc(Cn2cc(C(=O)C(=O)Nc3cc(C)ns3)c3ccccc32)on1** | **N#Cc1ccc(Cn2cc(C(=O)C(=O)Nc3cc(-c4ccc(Cl)cc4)no3)c3ccccc32)cc1** |
| **CHEMBL1153221** | **CHEMBL1071926** | **Cn1c(CN2CCN(c3ncc(Cl)cn3)CC2)nc2ccccc21** | **Cn1c(CN2CCC(c3nsc4ccccc34)CC2)nc2ccccc21** |
| **CHEMBL1140044** | **CHEMBL975409** | **Cn1cc(C(=O)O)c(=O)c2cc(N)c(N3CCN(c4ccccn4)CC3)cc21** | **Nc1cc2c(=O)c(C(=O)O)c[nH]c2cc1N1CCN(c2nc3ccccc3o2)CC1** |
| **CHEMBL4411320** | **CHEMBL4413800** | **O=c1ccn([C@@H]2O[C@H](CO)[C@@H](O)[C@@]2(F)Br)c(=O)[nH]1** | **Nc1ncnc2c1ncn2[C@@H]1O[C@H](CO)[C@@H](O)[C@@]1(F)Br** |
| **CHEMBL1255520** | **CHEMBL1259521** | **CN1CCc2sc(OCCCN3CCCCC3)nc2C1** | **c1ccc(-c2nc(OCCCN3CCCCC3)sc2-c2ccccc2)cc1** |
| **CHEMBL1141492** | **CHEMBL946123** | **CC(C)c1nc2ccccc2n1Cc1ccc(C(=O)N[C@@H]2CN(C(=O)OC(C)(C)C)C[C@@H]2C(=O)NO)cc1** | **CC(C)(F)c1nc2ccccc2n1Cc1ccc(C(=O)N[C@@H]2C[C@@]3(CCCO3)C[C@@H]2C(=O)NO)cc1** |
| **CHEMBL2062373** | **CHEMBL2066924** | **CN(C)C[C@@H](OC(=O)N1Cc2c(NC(=O)c3ccccc3)n[nH]c2C1(C)C)c1ccccc1** | **CN1CCN(C(=O)N2Cc3c(NC(=O)c4cccc(Oc5ccccc5)c4)n[nH]c3C2(C)C)[C@@H](Cc2ccccc2)C1** |
| **CHEMBL3580631** | **CHEMBL3583803** | **CC(=O)Nc1cc(S(=O)(=O)Nc2ccc(F)cc2F)cc2ccn(CCc3ccccc3)c12** | **O=C1CCCN1c1cc(S(=O)(=O)Nc2ccc(F)cc2F)cc2c1N(CCc1ccccc1)CC2** |
| **CHEMBL1833972** | **CHEMBL1837699** | **COCCCN1CCN(c2ncccc2Cn2c(=O)[nH]c3c(N)nc(C(F)(F)F)cc32)CC1** | **CN1CCN(CC2CCCN2c2cc(Cn3c(=O)[nH]c4c(N)nc(C(F)(F)F)cc43)ccn2)CC1** |
| **CHEMBL1145819** | **CHEMBL619854** | **CC1CCCN(CCC(C)N(C)S(=O)(=O)c2cccc3ccccc23)C1** | **CC1CCN(CC[C@@H]2CCCN2S(=O)(=O)c2cccc3ccccc23)CC1** |
| **CHEMBL4364257** | **CHEMBL4365089** | **CN(C)c1ccc(C(=O)NCCNC(=O)c2ccc(-c3ccc4c(N)n[nH]c4c3)cc2)cc1** | **Nc1n[nH]c2cc(-c3ccc(C(=O)N4CCN(C(=O)c5ccc(Cl)c(C(F)(F)F)c5)CC4)cc3)ccc12** |
| **CHEMBL1143527** | **CHEMBL833843** | **CC(=O)OC(c1ccc(C#N)cc1)c1cncn1Cc1ccc2c(c1)c(-c1cccc(Cl)c1)cc(=O)n2C** | **Cn1c(=O)cc(-c2cccc(Cl)c2)c2cc(Cn3cncc3Cn3ccc(C(=O)N4CCOCC4)c3)ccc21** |
| **CHEMBL2069278** | **CHEMBL2073239** | **Cn1c(=O)c2sc3sccc3c2c2cc(C3=NCCN3)ccc21** | **O=c1[nH]c2ccc(C3=NCCN3)cc2c2c1sc1cc(C3=NCCN3)ccc12** |
| **CHEMBL1135811** | **CHEMBL620617** | **O=S(=O)(c1ccccc1)C1CCN(CCc2ccc(F)cc2F)CC1** | **COc1ccc2c(c1)CCC1(CCN(CCc3ccc(F)cc3F)CC1)O2** |
| **CHEMBL3392965** | **CHEMBL3395231** | **O=C(c1cccc(N2CCCCC2)n1)N(C1CC1)[C@H]1CC[C@@H](O)CC1** | **O=C(N[C@H]1C2CC3CC1C[C@](O)(C3)C2)c1cccc(N2CCCCC2)n1** |
| **CHEMBL2169840** | **CHEMBL2172956** | **COc1cccc(CC(=O)N2CCc3cc(-c4cn(C)c5ncnc(N)c45)ccc32)c1** | **Nc1ncc(-c2ccncc2)c2scc(-c3ccc4c(c3)CCN4C(=O)Cc3ccccc3)c12** |
| **CHEMBL2169754** | **CHEMBL2173142** | **CC(C)c1cc(C(F)(F)F)c2nc(C(=O)N3CCC(N4CCOC4=O)CC3)c(Cl)n2c1** | **O=C(c1nc2c(C(F)(F)F)cc(-c3ccoc3)cn2c1Cl)N1CCC(c2cccs2)CC1** |
| **CHEMBL1148732** | **CHEMBL894342** | **Cc1ccc(-n2cc3c(n2)c(N)nc2ccccc23)cc1** | **COc1ccc(-n2cc3c(n2)c(NC(=O)c2ccccc2)nc2ccccc23)cc1** |
| **CHEMBL1137049** | **CHEMBL852182** | **CN(C)CCNC(=O)c1nc2cc(Cl)ccc2[nH]1** | **CN(C)c1cccc(-c2nc3cc(Cl)ccc3[nH]2)c1** |
| **CHEMBL1770132** | **CHEMBL1772682** | **O=C(CCc1nc(-c2ccc(O)cc2)no1)Nc1ccccc1C(=O)O** | **O=C(CCc1cc2n(n1)-c1ccc(O)cc1CC2)Nc1ccccc1C(=O)O** |
| **CHEMBL3425442** | **CHEMBL3428281** | **CC12Cc3[nH]nc(C(=O)Nc4cnn(Cc5ccccc5)c4)c3CC1C2** | **C[C@@]12Cc3[nH]nc(C(=O)Nc4cnn([C@H](c5ccccc5)[C@H]5CCCCS5(=O)=O)c4)c3C[C@@H]1C2(F)F** |
| **CHEMBL1139353** | **CHEMBL954163** | **O=C(NCCCCN1CCN(c2cccc(Cl)c2Cl)CC1)c1cc2ccccc2cn1** | **O=C1c2cc3ccccc3n2CCN1CCCCN1CCN(c2cccc(Cl)c2Cl)CC1** |
| **CHEMBL1149144** | **CHEMBL799784** | **CC1(C)CCC(C)(C)c2cc(C3CCCc4oc(/C=C/C(=O)O)nc43)ccc21** | **CC1(C)CCC(C)(C)c2cc(C3CCCc4oc(/C=C5/SC(=O)NC5=O)cc43)ccc21** |
| **CHEMBL4130560** | **CHEMBL4136260** | **COC(=O)c1ccc2c(c1)NC(=O)/C2=C(\Nc1ccc(N2C(=O)CC[C@@H]2CN(C)C)cc1)c1ccccc1** | **CN1CCN(CC(=O)N(C)c2ccc(N/C(=C3\C(=O)Nc4cc(-c5nnco5)ccc43)c3ccccc3)cc2)CC1** |
| **CHEMBL1268982** | **CHEMBL1273058** | **CC(C)(CO)N1CCN(C(=O)OC2([C@H]3CCC[C@@H](C4CC4)N3S(=O)(=O)c3ccc(Cl)cc3)CC2)CC1** | **O=C(OC1([C@H]2COC[C@@H](CC3CC3)N2S(=O)(=O)c2ccc(Cl)cc2)CC1)N1CCC2(CCN2)CC1** |
| **CHEMBL3259531** | **CHEMBL3268019** | **Nc1nccc(Oc2ccc(NS(=O)(=O)CC(=O)Nc3ccc(F)cc3)cc2F)c1Cl** | **Cc1ccc(NC(=O)C2CCCN(c3ccc(Oc4ccnc(N)c4Cl)c(F)c3)S2(=O)=O)cc1** |
| **CHEMBL1135647** | **CHEMBL644954** | **Clc1ccc2c(c1)C(N1CCN(c3ccccc3)CC1)=Nc1cccnc1N2** | **Cc1ccc2c(c1)C(NC1CCN(Cc3ccccc3)CC1)=Nc1cccnc1N2** |
| **CHEMBL3259563** | **CHEMBL3266136** | **Cc1cccc(-n2ncc3c(NC[C@H](C)NS(=O)(=O)c4c(C)cc(C)cc4C)cccc32)c1** | **Cc1cc(C)c(S(=O)(=O)NC(COc2cccc3c2cnn3-c2ccc(F)cc2)C(F)(F)F)c(C)c1** |
| **CHEMBL2429834** | **CHEMBL2433166** | **O=C(NO)c1ccc2c(c1)CCN(S(=O)(=O)c1ccc(C(F)(F)F)cc1)C2** | **O=C(NO)c1ccc2c(c1)CCN(C(=O)C1CCCCCC1)CC2** |
| **CHEMBL1147836** | **CHEMBL871260** | **CCOc1cccc2cc(C(=O)N3CCC[C@H]3CN3CCCC3)oc12** | **O=C(c1cnc2ccccc2c1)N1CCC[C@H]1CN1CCCC1** |
| **CHEMBL4011612** | **CHEMBL4012481** | **OC(c1cncnc1Nc1ccc(F)cc1)C1CCNCC1** | **CS(=O)(=O)N1CCC(C(O)c2cncnc2Sc2ccc(F)cc2)CC1** |
| **CHEMBL5126669** | **CHEMBL5131184** | **CC1(C)C(=O)N(C2CCCC2)c2nc(NC3CCN(S(C)(=O)=O)CC3)ncc21** | **CC1(C)Cc2cnc(Nc3ccc(S(N)(=O)=O)cc3)nc2N(C2CCCC2)C1=O** |
| **CHEMBL1125519** | **CHEMBL656568** | **NS(=O)(=O)c1cc2cc(CN3CCOCC3)sc2o1** | **NS(=O)(=O)c1cc2cc(CNCc3ccccn3)sc2o1** |
| **CHEMBL1777722** | **CHEMBL1781717** | **CNC(=O)c1cc(Oc2ccc3c(c2)nc(Nc2cccc(C(C)(C)C)c2)n3C)ccn1** | **CCc1ccc(Nc2nc3cc(Oc4ccnc(C(=O)NC)c4)ccc3o2)cc1** |
| **CHEMBL1153345** | **CHEMBL960918** | **CNc1nc2sc(-c3cccc(CNC(C)=O)c3)nc2c2c1ncn2C** | **CNc1nc2sc(C(=O)NC3CCCC3)nc2c2c1ncn2C** |
| **CHEMBL4428050** | **CHEMBL4428803** | **Nc1cc(C(F)(F)F)c(-c2cc(N3CCOCC3)nc(Nc3ccc(N4CCOCC4)cc3)n2)cn1** | **Nc1cc(C(F)(F)F)c(-c2nc(N3CCOCC3)nc(N3C[C@H]4C[C@@H]3CO4)n2)cn1** |
| **CHEMBL1821611** | **CHEMBL1826162** | **NC1=NC2(CCCCC2)N(OCc2ccccc2)C(N)=N1** | **COc1ccc(CN2C(N)=NC(N)=NC23CCCCC3)cc1** |
| **CHEMBL1128650** | **CHEMBL818492** | **O=C(O)CNC(=O)c1cc2n(n1)CCN(CCCC1CCNCC1)C2=O** | **O=C(O)CCNC(=O)c1cc2n(c1)CCN(CCC1CCNCC1)C2=O** |
| **CHEMBL3860088** | **CHEMBL3861541** | **Cc1cc(-c2ncc(CNC(=O)c3ccc4c(c3)oc3ccccc34)cc2F)ccn1** | **Cc1cc(-c2ccc(CNC(=O)c3ccc4c(c3)C(=O)c3ccccc3-4)cc2)ccn1** |
| **CHEMBL3638822** | **CHEMBL3707821** | **Cc1nc2ccc(F)cc2nc1-c1cc2nc(N3CC[C@H](F)C3)cc(N[C@H]3CC[C@@](C)(O)CC3)n2n1** | **Cc1nc(C)c(C2CC2)nc1-c1cc2nc(N3CCCC3)cc(NC3CCOCC3)n2n1** |
| **CHEMBL1132360** | **CHEMBL643527** | **CN(CC1CCN(c2ncccn2)CC1)CC1COc2ccccc2O1** | **c1cnc(NCC2CCN(CC3COc4ccccc4O3)CC2)nc1** |
| **CHEMBL5042504** | **CHEMBL5043883** | **CCCNNC(=O)c1ccc(CNC(=O)c2cc3cc(Br)ccc3[nH]2)cc1** | **CCCNNC(=O)c1ccc(CNC(=O)c2nc3ccccc3s2)cc1** |
| **CHEMBL2046386** | **CHEMBL2050455** | **CCCS(=O)(=O)Nc1ccc(F)c(C(=O)Nc2cnc3[nH]nc(OC)c3c2)c1F** | **CCCS(=O)(=O)Nc1ccc(F)c(NC(=O)Nc2ncnc3[nH]ncc23)c1F** |
| **CHEMBL1136829** | **CHEMBL643559** | **Nc1ncnc2nc(-c3ccc(N4CCC5(CC4)COCO5)nc3)cc(-c3cccc(Br)c3)c12** | **Nc1ncnc2nc(-c3ccc(N4CCC(=NOC5CCOCC5)CC4)nn3)cc(-c3cccc(Br)c3)c12** |
| **CHEMBL1772999** | **CHEMBL1775949** | **CCn1ccc2c(-c3cnc(N)nc3-c3ccccc3O)cccc21** | **Nc1ncc(-c2ccc(N3CCOCC3)cc2)c(-c2ccccc2O)n1** |
| **CHEMBL1125439** | **CHEMBL766698** | **Nc1nc2c(O)ncn(Cc3ccccc3)c-2n1** | **Nc1nc(O)c2nc(N)n(Cc3ccccc3)c2n1** |
| **CHEMBL4480380** | **CHEMBL4480708** | **C/C(=N\NC(=O)Nc1nc2ccc(CN3CCC(C)CC3)cc2s1)c1ccccc1** | **CC1CCN(Cc2ccc(NC(=O)NN=C(c3ccccn3)c3ccccn3)cc2)CC1** |
| **CHEMBL5338740** | **CHEMBL5340419** | **Cc1cc(-c2ccc(S(N)(=O)=O)cc2)cnc1-c1ccnc2ccccc12** | **NS(=O)(=O)c1ccc(-c2ccc(-c3ccnc4ccccc34)cn2)s1** |
| **CHEMBL4190408** | **CHEMBL4195906** | **COc1ccc(NC(=O)N2CCN(c3nc(N)nc4scnc34)CC2)cc1** | **COc1ccc(NC(=O)N2CCN(c3nc(N)nc4sccc34)[C@@H](C)C2)c(C)c1** |
| **CHEMBL1938349** | **CHEMBL1944111** | **OC1(c2ccc(Cl)cc2)CCN(CCCSc2ccc(F)cc2)CC1** | **Fc1ccc(OCCCN2CCN(c3ccc(Cl)cc3)CC2)cc1** |
| **CHEMBL1153406** | **CHEMBL1022423** | **Nc1ccccc1NC(=O)c1ccc(C(=O)Nc2cccc(Nc3ncc(-c4cccnc4)cn3)c2)cc1** | **Cc1ccc(NC(=O)c2ccc(C(=O)Nc3ccccc3N)s2)cc1Nc1ncc(-c2cccnc2)s1** |
| **CHEMBL3407425** | **CHEMBL3413248** | **O=C(Cn1c(=O)c(Cc2ccccc2)nc2ccccc21)NNC(=O)c1ccc(Br)cc1** | **O=C(NNc1nc2ccccc2nc1Cc1ccccc1)c1ccc(O)cc1** |
| **CHEMBL4422622** | **CHEMBL4423292** | **CC(C)C[C@H]1COc2cc(F)ccc2N1C(=O)c1ccc2c(c1)NC(=O)CC2** | **C[C@@H]1CCc2cc(F)ccc2N1C(=O)c1ccc2c(c1)NC(=O)CO2** |
| **CHEMBL1131240** | **CHEMBL615581** | **COc1ccccc1N1CCN(CCc2ccc3c(c2)OCC(=O)N3C)CC1** | **Cn1c(=O)sc2cc(CCN3CCN(c4ccccc4O)CC3)ccc21** |
| **CHEMBL1132638** | **CHEMBL617605** | **COc1ccc2c(c1)C(N1CCN(C)CC1)=Nc1ccccc1N2** | **CN1CCN(C2=Nc3ccccc3Oc3ccc(OS(C)(=O)=O)cc32)CC1** |
| **CHEMBL4680084** | **CHEMBL4681206** | **Nc1ccc(CNC(=O)N2C(=O)CC2CC(=O)O)cc1** | **Cc1ccccc1NC(=O)N1C(=O)CC1CC(=O)NCCC(=O)O** |
| **CHEMBL1140556** | **CHEMBL941667** | **CS(=O)(=O)c1ccccc1-c1ccc2c(c1)CCN2C(=O)c1cc(C(F)(F)F)nn1-c1ccc2onc(N)c2c1** | **CN(C)Cc1nccn1-c1ccc2c(c1)CCN2C(=O)c1cc(C(N)=O)nn1-c1ccc2onc(N)c2c1** |
| **CHEMBL5113482** | **CHEMBL5117713** | **N#Cc1cccc(NC(=O)c2[nH]cnc2-c2nc3ccccc3[nH]2)c1** | **O=C(c1[nH]cnc1-c1nc2ccccc2[nH]1)N1CCOCC1** |
| **CHEMBL1145400** | **CHEMBL889560** | **CCc1cccc(-c2[nH]c(CNc3ccc(C#N)cc3)nc2-c2ccc3c(c2)OCO3)n1** | **Cc1cccc(-c2[nH]c(CNc3cccc(C#N)c3)nc2-c2ccc3nccnc3c2)n1** |
| **CHEMBL4219241** | **CHEMBL4223975** | **Cc1ccc(-n2c(Cn3ccc4nc(-c5ccccc5F)nc-4c3)nc3ccccc32)cc1** | **O=c1ccccn1Cc1ccc(-n2cc(Cn3ccc4nc(-c5ccccc5F)nc-4c3)nn2)cc1** |
| **CHEMBL3792291** | **CHEMBL3795301** | **O=C(Nc1ccccc1)N1CCN(c2nc3c(c(N4CCOCC4)n2)CSCC3)CC1** | **O=C(Nc1ccc(-c2nc3c(c(N4CCOCC4)n2)CSCC3)cc1)NS(=O)(=O)c1ccc(Cl)cc1** |
| **CHEMBL1153504** | **CHEMBL1057310** | **O=c1c2c(C(F)(F)F)nccc2nc(-c2cscn2)n1CCc1ccccc1** | **C[C@H](Cc1ccccc1)n1c(-c2ncccc2O)nc2ccnc(C(F)(F)F)c2c1=O** |
| **CHEMBL1131411** | **CHEMBL679530** | **NC(=O)N1CCC(CC(=O)N2CCN(C3c4ccc(Cl)cc4CCc4cc(Br)cnc43)CC2)CC1** | **O=C(Cc1cc[n+]([O-])cc1)N1CCN(C2c3ccc(Cl)c(F)c3CCc3cc(Br)cnc32)CC1** |
| **CHEMBL2034977** | **CHEMBL2038828** | **CN1CCCN(C(=O)c2ccc(F)c(-c3ccc(/C=C4/C(=O)Nc5ccc(Cl)cc54)o3)c2)CC1** | **O=C1Nc2ccc(F)cc2/C1=C\c1ccc(-c2cccc(C(=O)NCCN3CCCC3)c2)o1** |
| **CHEMBL2203244** | **CHEMBL2216152** | **CC(=O)Nc1nc(C)c(-c2cnc(N3CCCC(O)C3)o2)s1** | **CC(=O)Nc1nc(C)c(-c2cnc(Oc3cccnc3)o2)s1** |
| **CHEMBL3421608** | **CHEMBL3424979** | **c1cc2c(-c3ccnc4[nH]ccc34)c[nH]c2cn1** | **CS(=O)(=O)c1ccc(-c2nccc(-c3c[nH]c4cnccc34)n2)cc1** |
| **CHEMBL2203077** | **CHEMBL2215644** | **COC(=O)c1c(CN2CC[C@@H](S(C)(=O)=O)C2)c(=O)c2ccc(F)cc2n1-c1ccccc1** | **COC(=O)c1c(Cc2ccc(S(C)(=O)=O)cc2)c(=O)c2ccc(C(F)(F)F)nc2n1-c1ccccc1** |


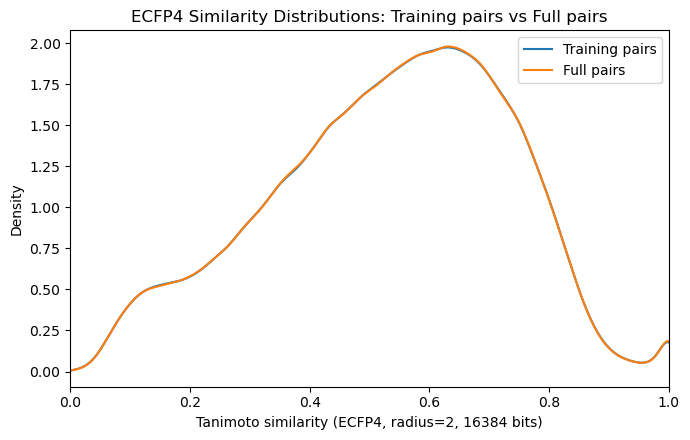


**Figure S1. ECFP4 similarity distributions of training pairs and full pairs.** ECFP4 similarity distributions of the pairs generated after molecule filtering process (orange line), together with the pairs used for model training (blue line). Pairwise similarities were computed as Tanimoto coefficients between RDKit Morgan (ECFP4; radius = 2) fingerprints with 16,384-bit length. The y-axis reports the estimated density of similarity values across pairs; in the full set (n = 608,642), 40,715 pairs (6.69%) have similarity < 0.2, while the training split shows a comparable fraction (32,646 / 486,916; 6.70%).

**
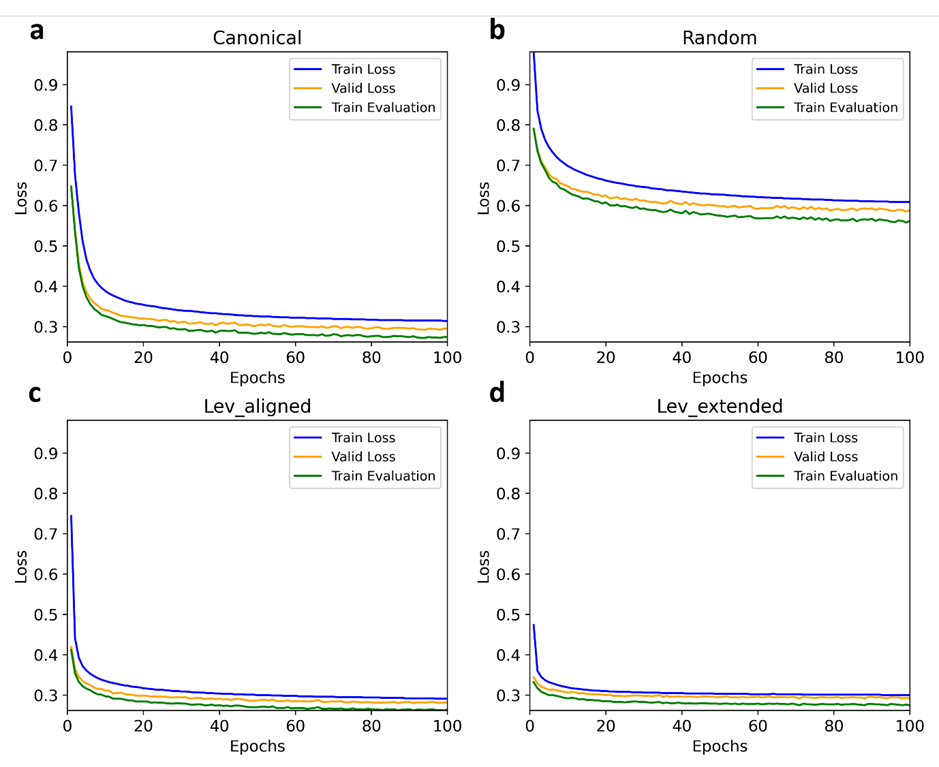
**

**Figure S2. Cross-entropy loss curves of different models during training and validation.** Cross-entropy loss curves of four trained models: (a) Canonical model, (b) Random model, (c) Lev_aligned model, and (d) Lev_extended model. The Training Loss (blue line) represents the cross-entropy loss calculated on the training dataset during model training. The Validation Loss (orange line) denotes the loss calculated on the validation dataset for evaluating the model's generalisation capability. The Training Evaluation Loss (green line) refers to the loss calculated on the entire training dataset at the end of each epoch, with weights fixed, to assess how well the model has already learned the training data before proceeding to the next epoch.

**
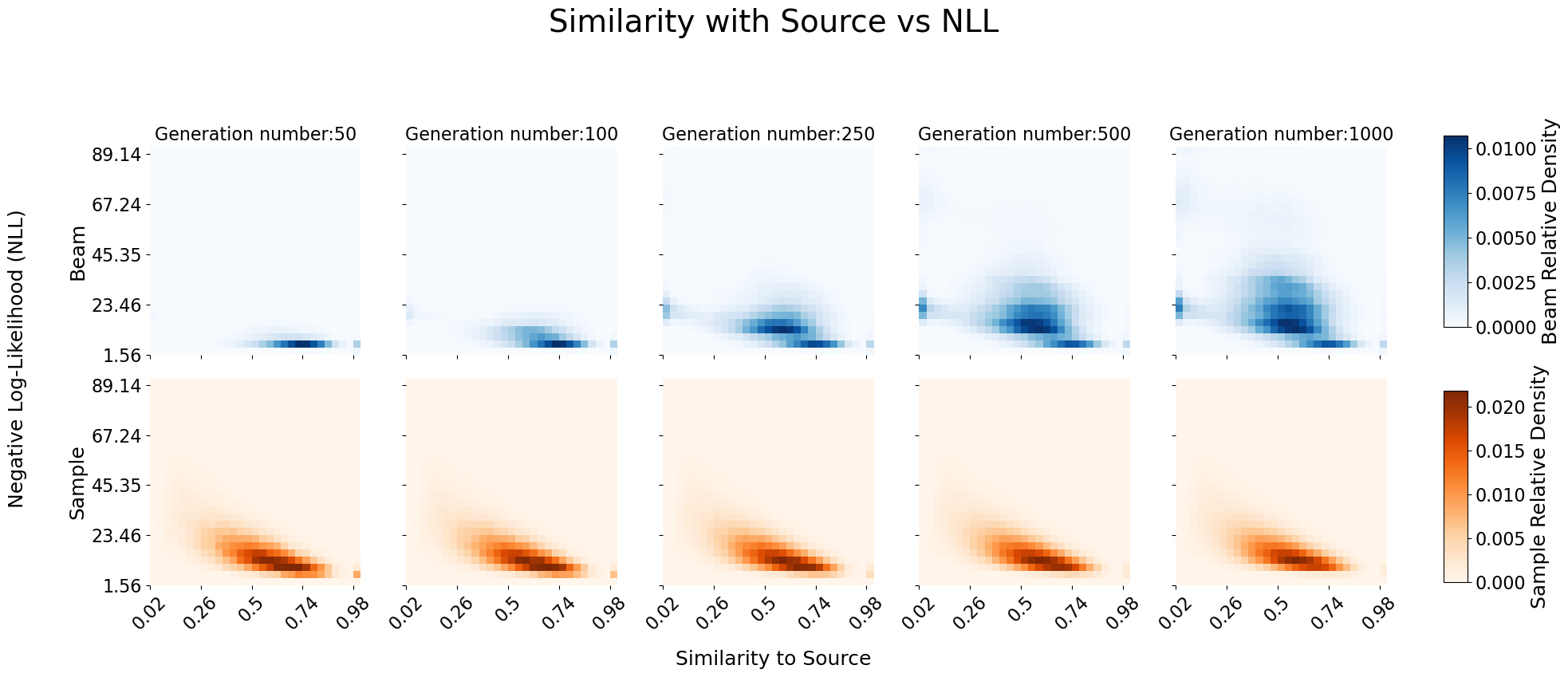
Figure S3. Similarity–Likelihood Density Heatmaps.** Density heatmaps illustrating the joint distribution of cumulative negative log-likelihood (NLL) and Tanimoto similarity to the source molecule for two generation methods (beam search in blue, multinomial sampling red) across five generation numbers (50, 100, 250, 500, 1000). Each column represents a fixed number of generated SMILES, with the top row showing beam search results and the bottom row showing multinomial sampling. Within each panel, the x-axis spans the similarity range (~0.02 to 0.98) and the y-axis the cumulative NLL (0 to 89.14), while the white-to-colour gradient encodes the normalized density of generated molecules in each similarity–NLL bin (deeper blue/red indicating higher density).

**
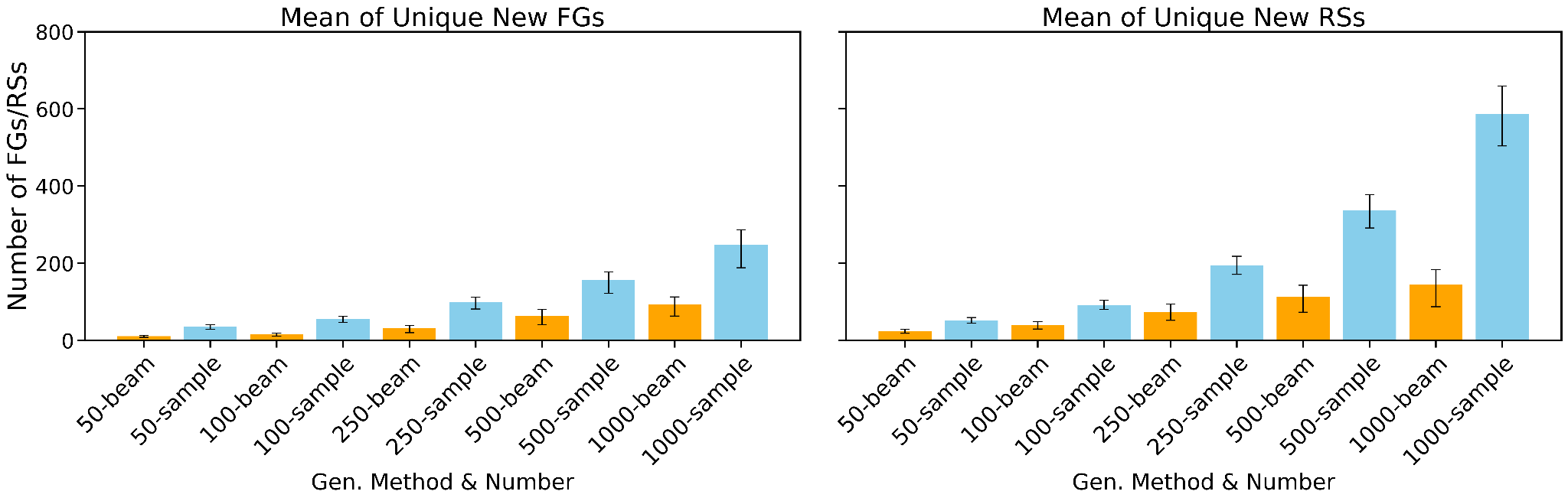
**

**Figure S4. Generation of Novel Structural Features.** Mean number of unique new (a) functional groups (FGs) and (b) ring systems (RSs) generated by ANNalog. From 1000 source-target pairs selected from ChEMBL35, 50, 100, 250, 500, and 1000 SMILES strings were generated per pair. Among the unique generated molecules, counts of new FGs and RSs were computed for each pair. Values are averaged across the 1000 pairs and grouped by generation method (beam search in orange, multinomial sampling in blue) and by the number of SMILES per pair. Error bars show the standard deviation across pairs.

**
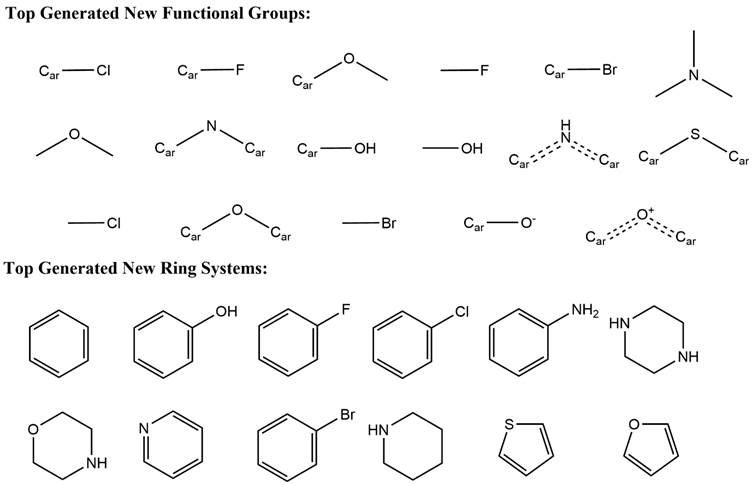
**

**Figure S5. Frequent New Functional Groups and Ring Systems.** Highly occurring new functional groups and ring systems introduced by ANNalog (both generation methods) across generated SMILES. In the functional group graph, "Cₐᵣ" denotes carbon atoms within aromatic ring systems. Only ring systems found within ChEMBL35 were included in this summary.


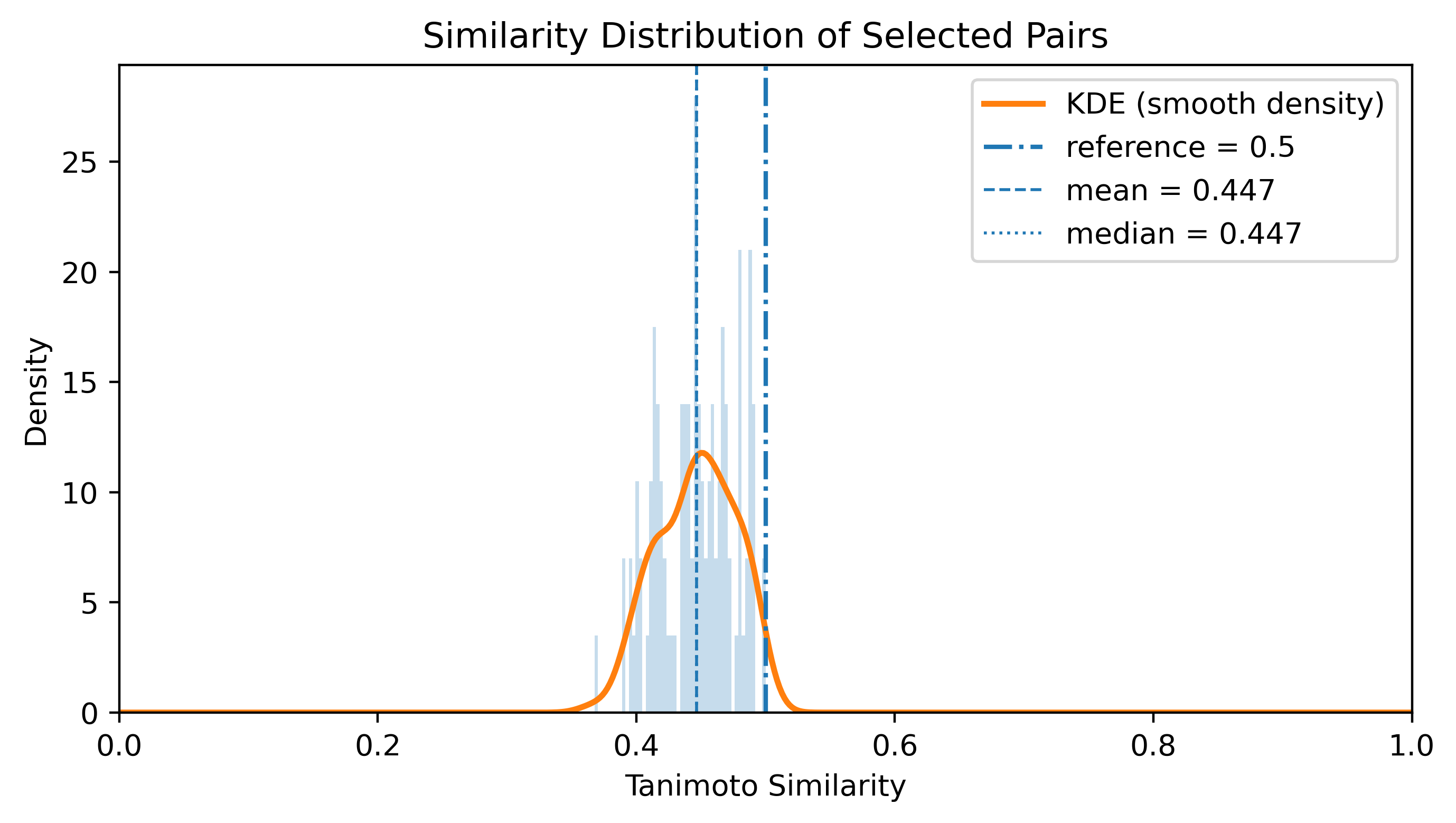


**Figure S6. Similarity Distribution of Selected Pairs.** Pairwise similarities between manually selected pairs were computed as the Tanimoto similarities between ECFP4 (Morgan radius = 2) fingerprints generated using RDKit with 16,384 bit length. The smooth curve represents a kernel density estimate of the similarity distribution (with a lightly shaded histogram shown for reference). Vertical dashed and dotted lines indicate the mean and median similarity, respectively, and the dash–dot line marks a reference similarity threshold of 0.5.

**
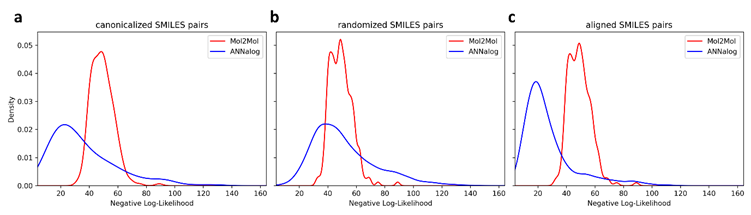
**

**Figure S7.** NLL Distributions Across SMILES Representations. Distributions of NLL values for canonicalised (a), randomized (b), and aligned (c) SMILES input pairs evaluated by the ANNalog (blue) and Mol2Mol (red) models.


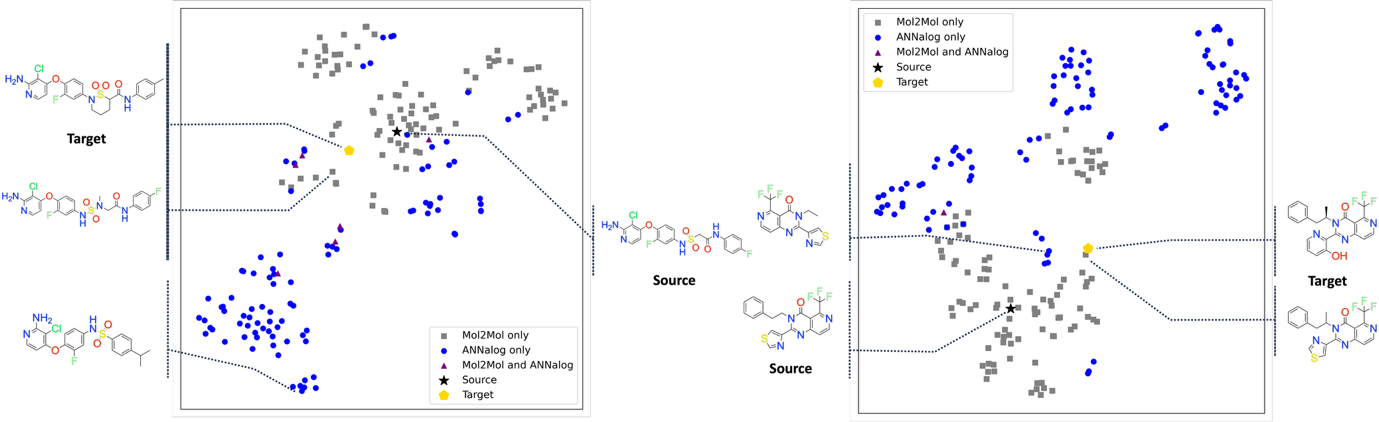


**Figure S8.** t-SNE Embeddings of Additional Generated Molecule Examples. t-SNE projections of ANNalog and Mol2Mol generated molecules for two additional source–target pairs, shown side by side, based on Morgan fingerprints and Jaccard distance. In each panel, the target is shown as a yellow pentagon, the source as a black star, ANNalog generated molecules as blue circles, Mol2Mol generated molecules as grey squares, and molecules generated independently by both models are shown as purple triangles. Highlighted structures include the generated molecule closest to the target and an additional representative molecule from a more distant region of the embedding space, illustrating alternative scaffold modifications.


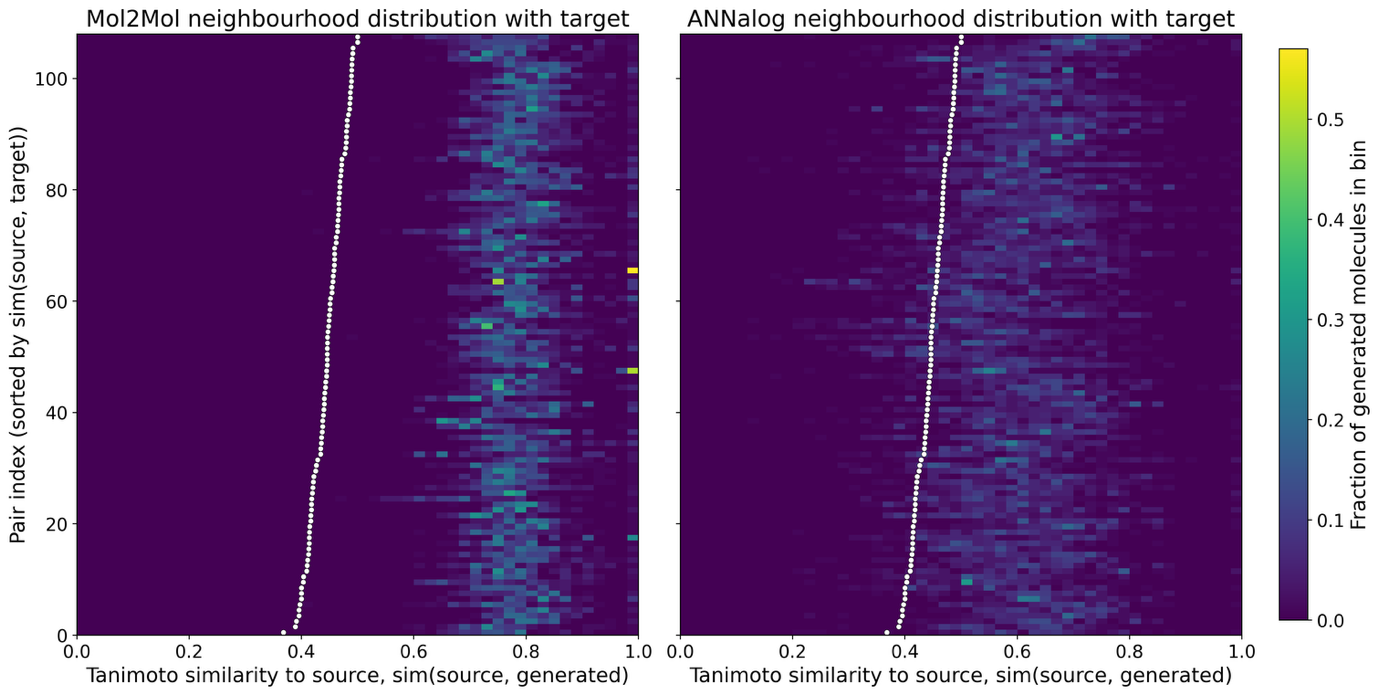


**Figure S9. Target position relative to generated neighbourhood similarities.** Heatmaps summarise, for each of the 108 curated source target pairs, the distribution of Tanimoto similarities between the source molecule and the set of generated molecules, sim(source, generated), for Mol2Mol (left) and ANNalog (right) under the same generation budget. For each pair, up to 100 unique molecules were generated per source using beam search. Each row corresponds to one source target pair and is plotted as a histogram of sim(source, generated) values binned from 0 to 1 (bin width 0.02), with colour indicating the fraction of generated molecules falling into each bin (row normalised so fractions sum to 1). Rows are ordered by sim(source, target) from low to high, using the same row ordering in both panels. Overlaid circle markers indicate sim(source, target) for the corresponding pair. Similarities were computed as Tanimoto coefficients of ECFP4 (Morgan radius 2) fingerprints with 16,384 bit length.
